# Transcriptome and hormone metabolome reveal the mechanism of stem bending in water lily (*Nymphaea tetragona*) cut-flowers

**DOI:** 10.3389/fpls.2023.1195389

**Published:** 2023-09-08

**Authors:** Jie Li, Yuhui Sheng, Huixian Xu, Qinxue Li, Xiuya Lin, Yang Zhou, Ying Zhao, Xiqiang Song, Jian Wang

**Affiliations:** ^1^ Key Laboratory of Genetics and Germplasm Innovation of Tropical Forest Trees and Ornamental Plants, Ministry of Education, College of Forestry, Hainan University, Haikou, Hainan, China; ^2^ Key Laboratory of Germplasm Resources Biology of Tropical Special Ornamental Plants of Hainan, Haikou, Hainan, China; ^3^ College of Agricultural, Hengxing University, Qingdao, Shandong, China

**Keywords:** water lily, cut-flowers, stem bending, transcriptome, hormone level, paraffin section

## Abstract

Water lilies are popular ornamental cut-flowers with significant economic and cultural value. However, stem bending affects the preservation of cut-flowers during their vase life. To gain further insights into the molecular mechanisms of stem bending, transcriptome profiling, hormone measurement, and morphological analysis were performed using the stems of the ‘Blue Bird’ water lily. Transcriptome analysis revealed that 607 differentially expressed genes (DEGs) were associated with the dorsal and ventral stems of the water lily, of which 247 were up-regulated and 360 were down-regulated. Significant differences in genes associated with plant hormones, calcium ions, glucose metabolism, and photosynthesis pathways genes involved in the dorsal and ventral areas of the curved stem. In particular, DEGs were associated with the hormone synthesis, gravity response, starch granules, Ca^2+^ ions, and photosynthesis. The results of qRT-PCR were consistent with that of the transcriptome sequence analysis. A total of 12 hormones were detected, of which abscisic acid, indole-3-carboxaldehyde, indole-3-carboxaldehyde and jasmonic acid were significantly differentially expressed in the dorsal and ventral stems, and were significantly higher in the dorsal stem than in the ventral stem. The cell morphology in the dorsal and ventral areas of the curved stem clearly changed during vase life. The direction of starch granule settlement was consistent with the bending direction of the water lily stem, as well as the direction of gravity. In conclusion, stem bending in water lily cut-flowers is regulated by multiple factors and genes. This study provides an important theoretical basis for understanding the complex regulatory mechanism of water lily stem bending.

## 1 Introduction

Water lilies (*Nymphaea tetragona*, *Nymphaea* L.) are a perennial aquatic flower plant of the order Nymphaeaceae, and have high ornamental, edible, and medicinal value (Yu et al., 2018). Based on the adaptability of water lilies to climatic conditions, the Nymphaea water lily may be divided into two ecological types: tropical water lily and hardy water lily (Xie et al., 2022). Water lilies are known as the “sleeping beauty of flowers”. They have colorful and fragrant flowers with numerous petals and long stalks, with considerable potential to be used as cut-flowers (Samarakoon, 2005). However, stem bending is one of the primary factors affecting the preservation of cut-flowers during their vase life. Thus, it is important to identify the mechanism of water lily stem bending to prolong the vase life of water lilies and improve their ornamental value.

The gravitropic response is a dynamic process in which plants perceive gravitropic stimuli and change their growth direction during development. This process may be divided into four stages, including the sensing of gravitropic signals, transmission of gravitropic signals, asymmetric distribution of auxin, and curved growth of gravitropic responsive organs (Strohm et al., 2012; Wu et al., 2016; Wang et al., 2018). Sensing and backward transmission of gravity signals into second messengers occur by signaling molecules such as Ca^2+^, InsP3, and PIN. Subsequent asymmetric transport of auxin transmits these signals to various parts of the gravity responsive organs, which results in differences in downstream substrate responses, such as curved growth of the gravity responsive organs (Strohm et al., 2012; Baldwin et al., 2013). Thus, proper bending requires the coordination of various cellular processes, including signal transduction, plant hormone transport, and cell expansion, of which multiple genes are involved (Strohm et al., 2012; Eng and Sampathkumar, 2020; Jonsson et al., 2023).

Studies have confirmed that curved growth caused by gravity is the result of the asymmetric growth of plant organs (Strohm et al., 2012; Jonsson et al., 2023). When plant organs are stimulated by gravity, the amyloplast shifts and converts the physical signal into a biological signal. A previous study showed that *lazy2* regulates the rice tiller angle by specifically regulating starch biosynthesis in gravity sensing cells (Huang et al., 2021). Ca^2+^ acts as a signaling molecule, and Ca^2+^ channels are opened to transmit information from the sedimenting amyloplasts within the gravity-sensing cells (Nakamura et al., 2019). Studies have shown that cytoplasmic free Ca^2+^ concentrations increase in the petiole and hypocotyl of Arabidopsis seedlings expressing apoaequorin during gravity stimulation (Toyota et al., 2008; Toyota and Gilroy, 2013; Tatsumi et al., 2014). Ca^2+^ content is closely related to the flexibility of the peonies peduncle, which is an important indicator of the quality of cut-flowers. Calcium chloride treatment significantly improved the mechanical strength of peonies peduncle (Li et al., 2012). Plants use signaling molecules to transmit gravity signals to phytohormones, which mediate the gravity bending response. Studies have shown that various hormones, such as auxin, ethylene, gibberellin, brassinosteroid (BR), jasmonic acid (JA), abscisic acid (ABA), and cytokinin (CK), are involved in this process; however, auxin plays a leading role (Mazzella et al., 2014; Gerttula et al., 2015; Ajala and Hasenstein, 2022; Wang et al., 2023). Plant hormones can be transported between plant tissues (Philosoph-Hadas et al., 2005) and accumulate in target tissues, which results in their asymmetric distribution (Friml, 2003). Several studies have indicated that ABC transporters function in the auxin transport phase of gravitropism (Lewis et al., 2007; Strohm et al., 2012). In addition to gravity, several environmental factors affect the growth direction of plants, such as nutrients, light, temperature, water, and minerals, which interact with gravity to regulate the growth and development of plants (Song et al., 2019). The expression of the *rice morphology determinant* gene has been reported to be inhibited by the phytochrome-interacting factor-like protein OsPIL16, and the overexpression of OsPIL16 resulted in gravisensing and actin patterning defects (Song et al., 2019).

Currently, the molecular mechanism of stem bending during the vase life of water lily cut-flowers is unknown. Therefore, we conducted transcriptomic analysis, hormone measurements, and morphological observations to examine stem bending in the dorsal and ventral stems of the water lily ‘Blue bird’. The curved stems of water lily cut-flowers were analyzed at the molecular, hormone metabolism, and cellular levels to understand and address the stem bending problem.

## 2 Materials and methods

### 2.1 Plant material

**Figure 1:**
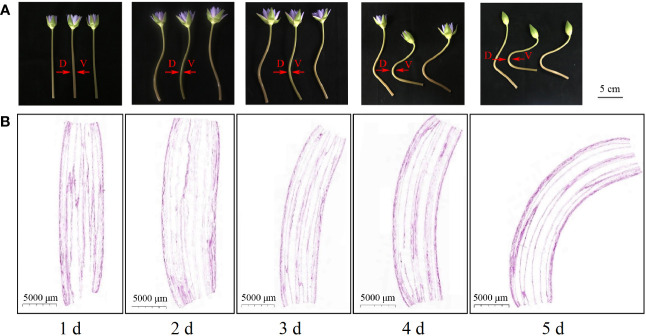
Phenotypes and longitudinal section of ‘Blue bird’ water lily flower stems during vase life. **(A)** phenotypes of the ‘Blue bird’ water lily stem, bar represents 5 cm. **(B)** longitudinal section of ‘Blue bird’ water lily flower stems, bar represents 5000 μm. D: dorsal stem; V: ventral stem.

Water lily (Nymphaea ‘Blue Bird’) plants were collected from DaDao lake, DaZhiPo town, Haikou city, Hainan, P.R. China (19.82 N, 110.63 E). The water lilies, which were collected on the first day of opening, exhibited erect stems and a largely consistent growth status. The pedicels were cut to a length of 25 cm, and inserted into a bottle (height, 22 cm) filled with ddH_2_O water. After the stems of cut-flowers were naturally bent on the fifth day, the bent area was cut with a scalpel and bisected into two parts by length cutting: the dorsal and ventral stems (**Figure 1A**). Some of the samples were used for cell morphology observation, whereas the remaining samples were rapidly frozen in liquid nitrogen and stored at −80°C for subsequent transcriptome analysis and hormone measurements. All RNA-seq and hormone analyses of the dorsal and ventral stems were performed in triplicate.

### 2.2 Paraffin section observation

Samples of the dorsal and ventral bent stems of ‘Blue bird’ water lily were fixed in FAA (37% formaldehyde, glacial acetic acid, 70% ethanol; 1:1:18 v:v:v) (Johansen, 1940). The FAA-fixed samples were dehydrated and embedded in paraffin using an embedding machine (JB-L5, Junjie Electronics Co., Ltd, Wuhan, China). Samples were sliced from the modified tissue wax block using a paraffin slicer (RM2016, Leica Instrument Co., Ltd, Shanghai, China). Dewaxing was performed by rinsing the sections in the following solutions: xylene I for 20 min, xylene II for 20 min, 100% ethanol I for 5 min, 100% ethanol II for 5 min, 75% ethanol for 5 min, and tap water. The sections were stained with PAS (G1008, Servicebio, Wuhan, China) dye solution B for 10–15 min, rinsed with distilled water, and with PAS A for 25–30 min in the dark. After rinsing for 5 min in tap water, the slides were stained with PAS C for 30 s and rinsed with tap water. The slices were subsequently treated with hydrochloric acid solution and ammonia, and washed with water between steps. Dehydration was performed using100% ethanol I for 5 min, 100% ethanol II for 5 min, 100% ethanol III for 5 min, xylene I for 5 min, and xylene II for 5 min. The samples were sealed with neutral gum (Sinopharm Chemical Reagent Co., Ltd). Finally, the cell morphology was observed via light microscopy using a NIKON ECLIPSE E100 microscope. Images were visualized using the NIKON DS-U3 imaging system.

### 2.3 Total RNA isolation and illumina sequencing

Total RNA of stems on the fifth day was extracted using a Trizol reagent, and mRNA was enriched using Oligo (dT) beads (Invitrogen, CA, USA). The enriched mRNA was fragmented into short fragments using a fragmentation buffer and reverse transcribed into cDNA with random primers. Second-strand cDNA was synthesized by adding DNA polymerase I, RNase H, dNTP, and buffer. The cDNA fragments were purified using the QiaQuick PCR extraction kit, repairing the ends, adding poly (A), and ligating to Illumina sequencing adapters. Finally, the ligation products were size selected by agarose gel electrophoresis, and PCR amplified. RNA-seq was performed using an Illumina HiSeq platform by Mega Genomics Health Science and Technology (Beijing) Co., Ltd. (Beijing, China).

### 2.4 Sequence filtration, assembly, unigene expression analysis and functional annotation

Raw reads obtained from the Illumina sequencing analysis were filtered to obtain high quality clean reads by removing reads containing adapters, 10% of the unknown nucleotides and >40% of low-quality (Q-value ≤ 10) bases. Transcriptome assembly of the clean reads from all sequenced samples was performed using Trinity (Grabherr et al., 2011). Firstly, the sequencing reads are fragmented into shorter fragments K-mers, then extends these small fragments into longer contiguous sequences Contigs, and utilizes the overlaps between these segments to generate a collection of fragments called Components. Finally, employing the De Bruijn graph method and sequencing read information, transcript sequences are identified within each fragment collection. The expression level of each transcript was calculated and normalized to FPKM (expected number of Fragments Per Kilobase of transcript sequence per Millions base pairs sequenced) (Trapnell et al., 2010). The default parameter of Subread package featureCounts 2.0.3 is used to calculate the FPKM quantitative calculation of the gene (Liao et al., 2014). DESeq (http://www.bioconductor.org/packages/release/bioc/html/DESeq.html) package was used to identify transcripts with a fold-change≥2, fold-change ≤ 0.5 and an *FDR*<0.01 in the dorsal and ventral stems were considered differentially expressed genes (DEGs). The identified genes were annotated using the following databases: NCBI non-redundant protein (NR), Swiss-Prot protein, Kyoto Encyclopedia of Genes and Genomes (KEGG), euKaryotic orthologous groups of proteins (KOG), and gene ontology (GO) using the BLAST (http://blast.ncbi.nlm.nih.gov/Blast.cgi) program with an e-value ≤ 10^−5^ (Conesa et al., 2005).


$$\text{FPKM}=\frac{\text{mapped fragments of transcript}}{\text{Total Count of mapped fragments }\left(\text{Millions}\right)\times \text{Length of transcript }\left(\text{kb}\right)}$$

### 2.5 qRT-PCR validation

Total RNA was isolated from the dorsal and ventral stems of the bent water lily stems using a Plant RNA Kit (R6827, Omega) according to the manufacturer’s protocol. The integrity of the total RNA was assessed on 1% agarose gels (**Figure S1**). Primer sequences for the selected DEGs (**Table S1**) were designed using the NCBI primer blast program (https://www.ncbi.nlm.nih.gov/tools/primer-blast/) and Primer Premier 5.0. The primers were synthesized by Sangon Biotech Co., Ltd. (Shanghai, China). Reverse transcription was performed using the PrimeScript RT kit with gDNA Eraser (TaKaRa, Shanghai, China) based on the manufacturer’s protocol. qRT-PCR was performed with three technical and three biological replicates. qRT-PCR analysis was performed using Luna Universal qPCR Master Mix (New England Biolabs, Ipswich, MA) according to the manufacturer’s instructions with denaturation at 95°C for 60 s and 40 cycles of amplification (95°C for 15 s and 60°C for 30 s). *Actin* was used as a reference control. The 2^−^ *^△△^* ^Ct^ method (Ct, cycle threshold value of target gene) was used to calculate relative mRNA expression levels (Willems et al., 2008).

### 2.6 Hormones identification and quantification

#### 2.6.1 Sample preparation and extraction

Plant materials (120 mg fresh weight) were ground to a fine powder in liquid nitrogen, extracted with 1.2 mL 80% methanol by vortexing briefly, and incubated for 16 h at 4°C. The extract was centrifuged for 15 min (12,000 g, 4°C) to remove solid particles. The resulting supernatant was evaporated to dryness under nitrogen gas stream, and reconstituted in 30% methanol (Li et al., 2014). The solution was centrifuged for 15 min (12,000 g, 4°C) and the supernatant was analyzed by LC-MS.

#### 2.6.2 LC/MS/MS analysis

The extracts were analyzed using an LC-ESI-MS/MS system (Ultra Performance Liquid Chromatography, UPLC, Shim-pack UFLC SHIMADZU CBM30A system; Tandem mass spectrometry, MS/MS, Applied Biosystems 6500 Quadrupole Trap). Hormone separation was performed with a HPLC Waters ACQUITY UPLC HSS T3 C18 column (1.8 µm, 2.1 mm × 100 mm) using UP water with 0.04% acetic acid (solvent A) and acetonitrile with 0.04% acetic acid (solvent B) as mobile phases. The following parameters were used for HPLC: flow rate of 0.35 mL/min; column temperature 40°C; 5 μL injection; method: 0 min (95% A: 5% B), 0–11 min linear gradient to 5% A:95% B, 11–12 min (5% A: 95% B), 12–12.1 min (95% A: 5% B), and 12.1–15 min (95% A:5% B). The effluent was alternatively connected to an ESI-triple quadrupole-linear ion trap (Q TRAP)-MS. The ESI source operation parameters were as follows: ion source, turbo spray; source temperature, 500°C; ion spray voltage (IS), 5500 V; curtain gas, 35.0 psi; and collision gas medium. DP and CE for individual MRM transitions were performed with further DP and CE optimization. A specific set of MRM transitions was monitored for each period according to the plant hormones eluted during this period.

### 2.7 Statistical analysis

All measurements and qRT-PCR analyses were performed in triplicate. Univariate values were analyzed using ANOVA and mean values were compared using Duncan’s new multiple range test (*P<* 0.05) with SPSS 22.0 software (IBM Corporation, United States).

## 3 Results

### 3.1 Microscopic observation of cut-flowers

#### 3.1.1 Observation of longitudinal flower stem sections during vase life

Paraffin sections of the longitudinal stem samples of the ‘Blue bird’ water lily cut-flowers (**Figure 1B**) revealed that the pedicels were upright on the first day during vase life. The pedicel appeared to show a trend toward bending on the second day, but the bending angle was small. The cut-flower stems were significantly bent on the third day compared with the first day. The flower stem bending angle continued to increase on the fourth and fifth day, and was the greatest on the fifth day. The results indicated that the cut-flower stems were clearly bent on the third day during vase life. With an increase in time, the level of the bending of the cut-flowers increased.

#### 3.1.2 Cell morphology of the dorsal and ventral stems

**Figure 2:**
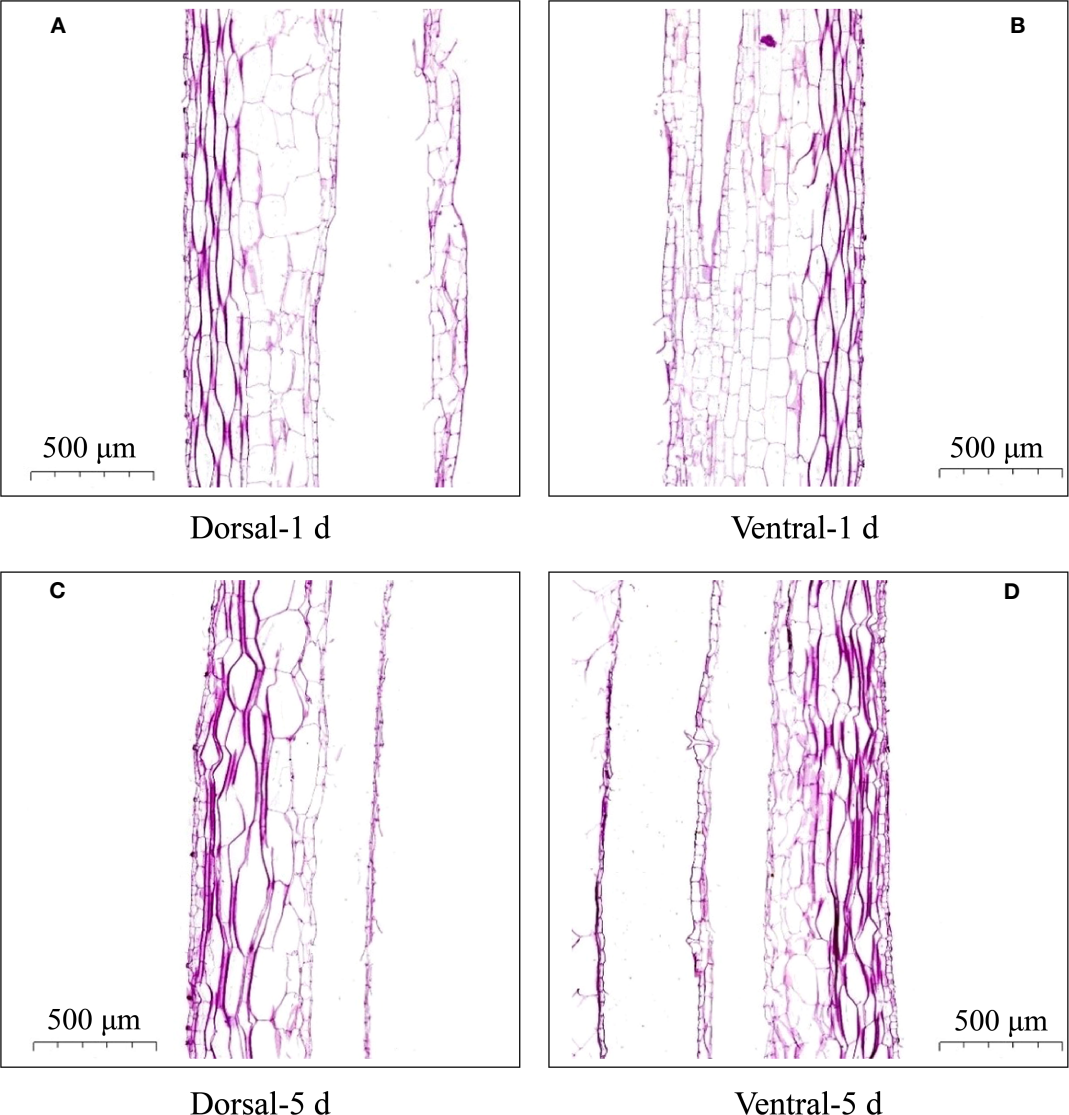
Cell morphology of the dorsal and ventral stems of the ‘Blue bird’ water lily. **(A)** dorsal stem on the first day, **(B)** ventral stem on the first day, **(C)** dorsal stem on the fifth day, **(D)** ventral stem on the fifth day.

Microscopy of the dorsal and ventral stem cells of cut-flowers on the first- and fifth-days during vase life revealed no significant differences in the morphology of cells (**Figures 2A, B**). However, the dorsal stem cells were larger, and the ventral cells were smaller on the fifth day (**Figures 2C, D**).

#### 3.1.3 Starch granules of the dorsal and ventral stems

**Figure 3:**
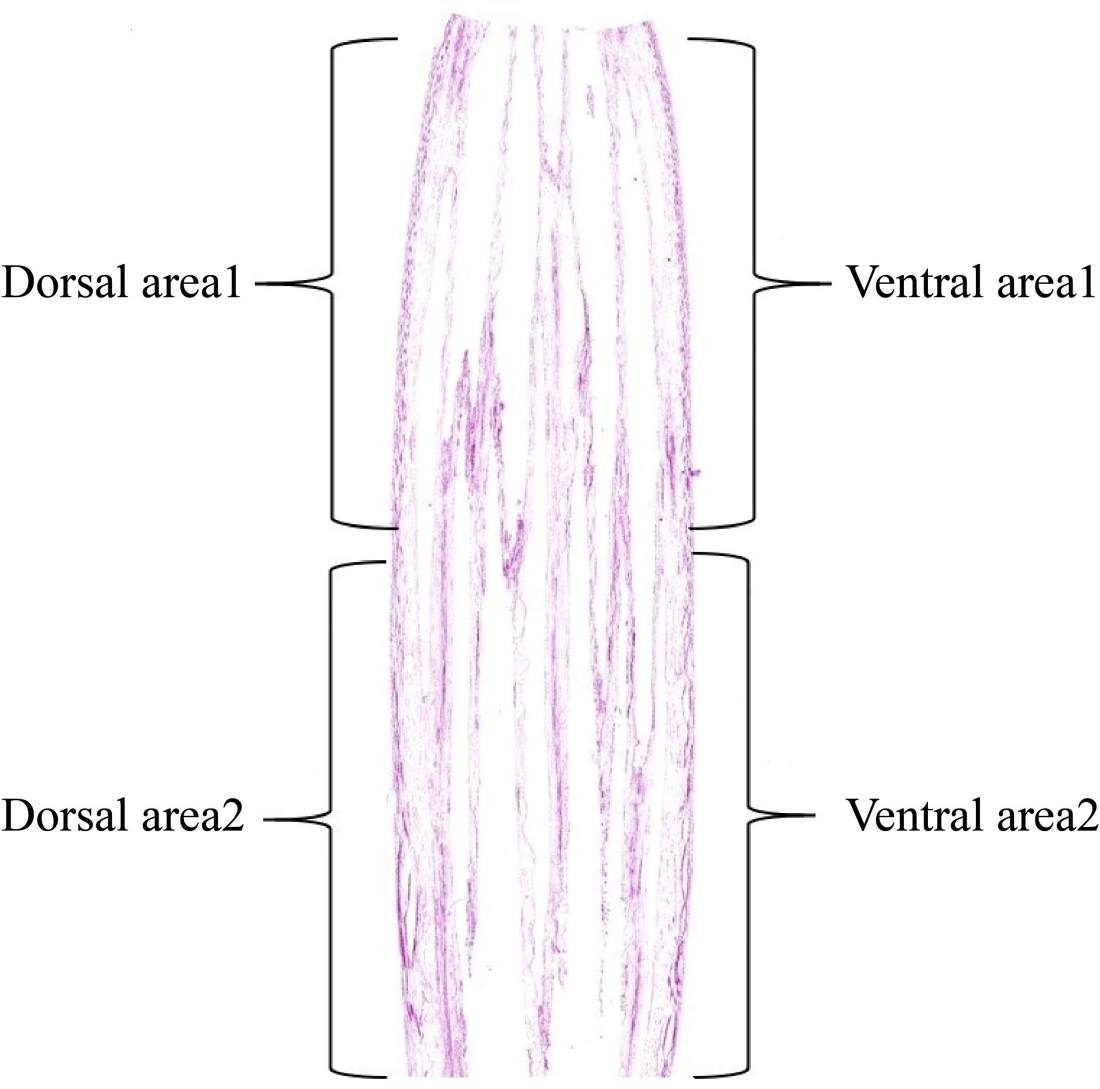
Division of the stem area in the ‘Blue bird’ water lily.

**Figure 4:**
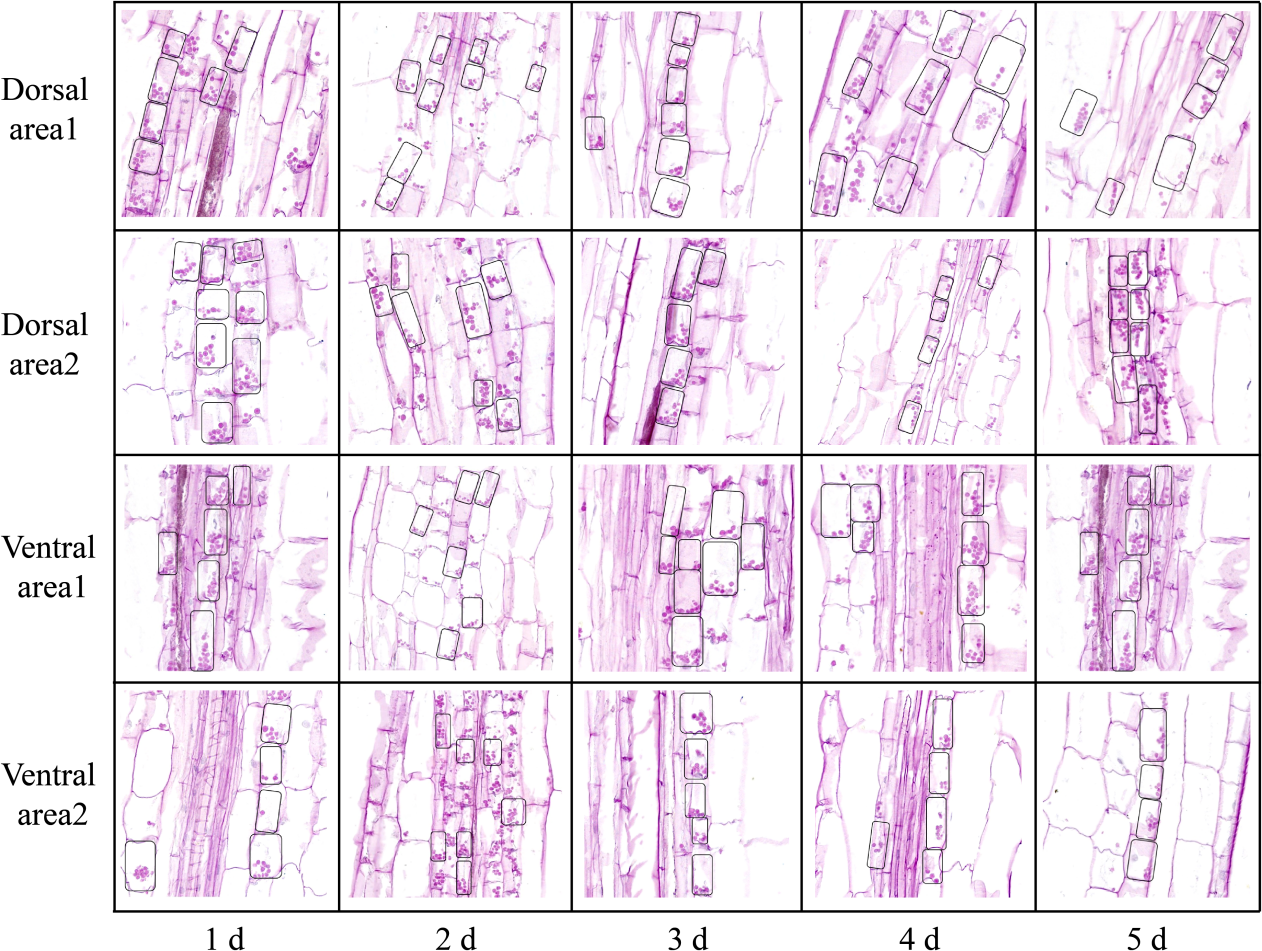
The intracellular distribution of starch granules in different areas of the dorsal and ventral stems of the ‘Blue bird’ water lily. The small black boxes are starch granules.

Examination of the starch granules revealed that there were a large number on the dorsal and ventral stems of the water lilies during vase life (**Figure 3**). The distribution of starch granules on the dorsal and ventral stems on the first day was similar and primarily on the left side or below the cell (**Figure 4**). The distribution of starch granules on the dorsal and ventral stems changed over time. On the second day, the distribution of starch granules was different between the dorsal and ventral stems; however, the distribution of starch granules on the dorsal areas 1 and 2, and ventral areas 1 and 2 was essentially the same (**Figure 4**). On the third day, the distribution of starch granules in different areas of the dorsal and ventral stems was relatively concentrated (**Figure 4**). On the fourth day, the distribution of starch granules in different areas of the cell continued to change (**Figure 4**). By the fifth day, the starch granules were mostly concentrated on the right side of the cell (**Figure 4**).

**Figure 5:**
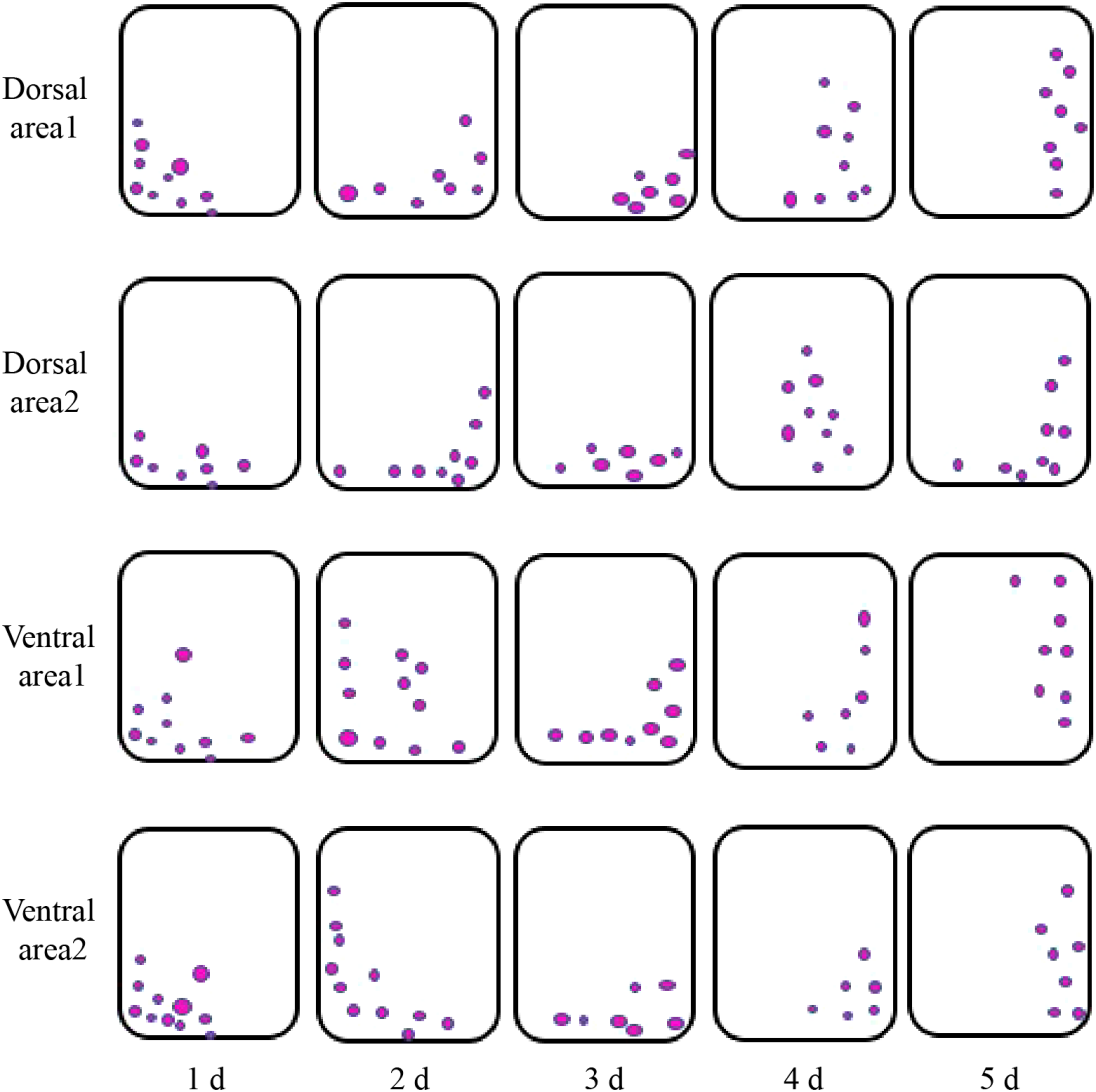
Schematic representation of the intracellular distribution of starch granules in different areas of the dorsal and ventral stems of the ‘Blue bird’ water lily.

**Figure 5** shows a schematic representation of the intracellular distribution of starch granules in the dorsal and ventral stems of the ‘Blue bird’ water lily cut-flowers. The starch granules distributed in the cells exhibited a 90° clockwise rotation from the first day to the fifth day during vase life, and the direction of rotation was consistent with the bending direction of the water lily stem (**Figure 5**). The intracellular distribution of starch granules in the ventral stems from the second day onward was different from that in the dorsal stems. Overall, a 90° clockwise rotation occurred in the intracellular distribution of starch granules from the first to fifth day, which was consistent with the direction in which the stems appeared to bend (**Figure 5**).

### 3.2 RNA sequencing, gene functional annotation, and classification

To analyze the gene expression profiles in the dorsal and ventral stems of the ‘Blue bird’ water lily during stem bending, RNA-seq was performed using the Illumina platform. A total of six cDNA libraries, three from the dorsal stems and three from the ventral stems were constructed and sequenced. A total of 41.05 GB of clean data were obtained, and the ratios of Q20, Q30, and GC contents were greater than 97.77%, 94.23%, and 48.16%, respectively (**Table S2**). A total of 214.53 million reads were mapped. The proportion of reads that were mapped to unique locations and multiple locations in the genome were 0.06%–0.07% and 76.54%–79.61%, respectively (**Table S3**). This indicates that the quality of the transcriptome data for each sample was high and confirmed that our transcriptome data were accurate and reliable.

A total of 95,337 unigenes were generated, of which 42,866 were annotated against public databases including COG, GO, KEGG, KOG, Pfam, Swiss-Prot, eggnog, and NR, and 10,568, 25,432, 24,386, 26,807, 38,156, and 40,871 genes were annotated using the COG, KOG, Pfam, Swiss-Prot, eggnog, and NR databases, respectively. GO annotations (11,685 genes) and KEGG pathway annotations (14,902 genes) were also obtained to gain more insights into putative gene function (**Table S4**, **Figures S2**, **S3**).

### 3.3 Identification of DEGs

**Figure 6:**
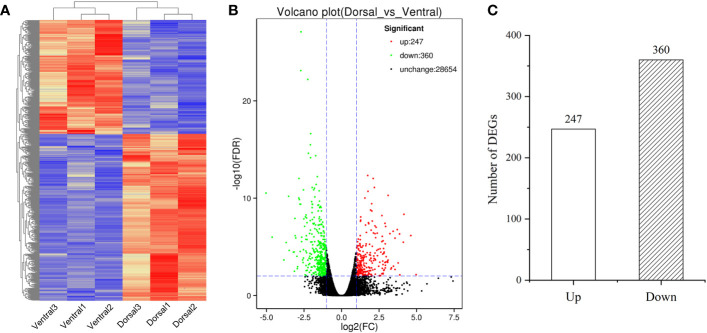
Transcriptomics analysis of the dorsal and ventral stems associated with ‘Blue bird’ water lily stem bending. **(A)** Heatmap of all DEGs. **(B)** Volcano plot of all DEGs with red representing the up-regulated genes, green representing the down-regulated genes, and black representing the genes that did not significantly change. **(C)** The number of up- and down-regulated genes in the Dorsal_vs_Ventral.

**Figure 7:**
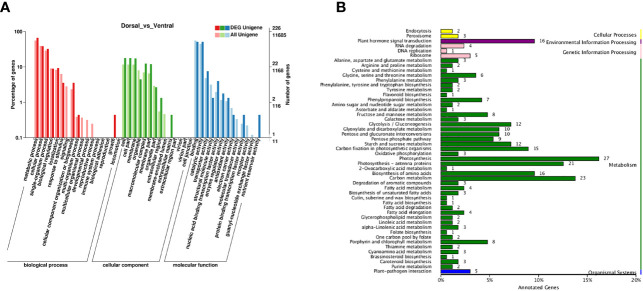
Enrichment analysis of DEGs. **(A)** GO enrichment analysis. The red, green, and blue columns indicate biological process, cellular components, and molecular function, respectively. **(B)** KEGG pathway enrichment analysis.

The DEGs in the comparison group Dorsal_vs_Ventral were analyzed. From the clustering heatmap in **Figure 6A**, it can be observed that the sample replicates exhibit good reproducibility. And 607 DEGs were identified, including 247 up-regulated genes and 360 down-regulated genes (**Figures 6B, C**, **Table S5**), of which 226 DEGs were annotated in the GO database. GO analysis, revealed the terms metabolic process, catalytic activity, cellular process, binding, and single-organism process as the top five annotations containing the largest number of genes (**Figure 7A**). KEGG analysis indicated that 212 DEGs were significantly associated with 61 metabolic pathways. The top five metabolic pathways were photosynthesis (27), carbon metabolism (23), photosynthesis-antenna proteins (21), plant hormone signal transduction (16) and amino acid biosynthesis (16) (**Figure 7B**).

### 3.4 DEGs involved in hormone signaling

**Figure 8:**
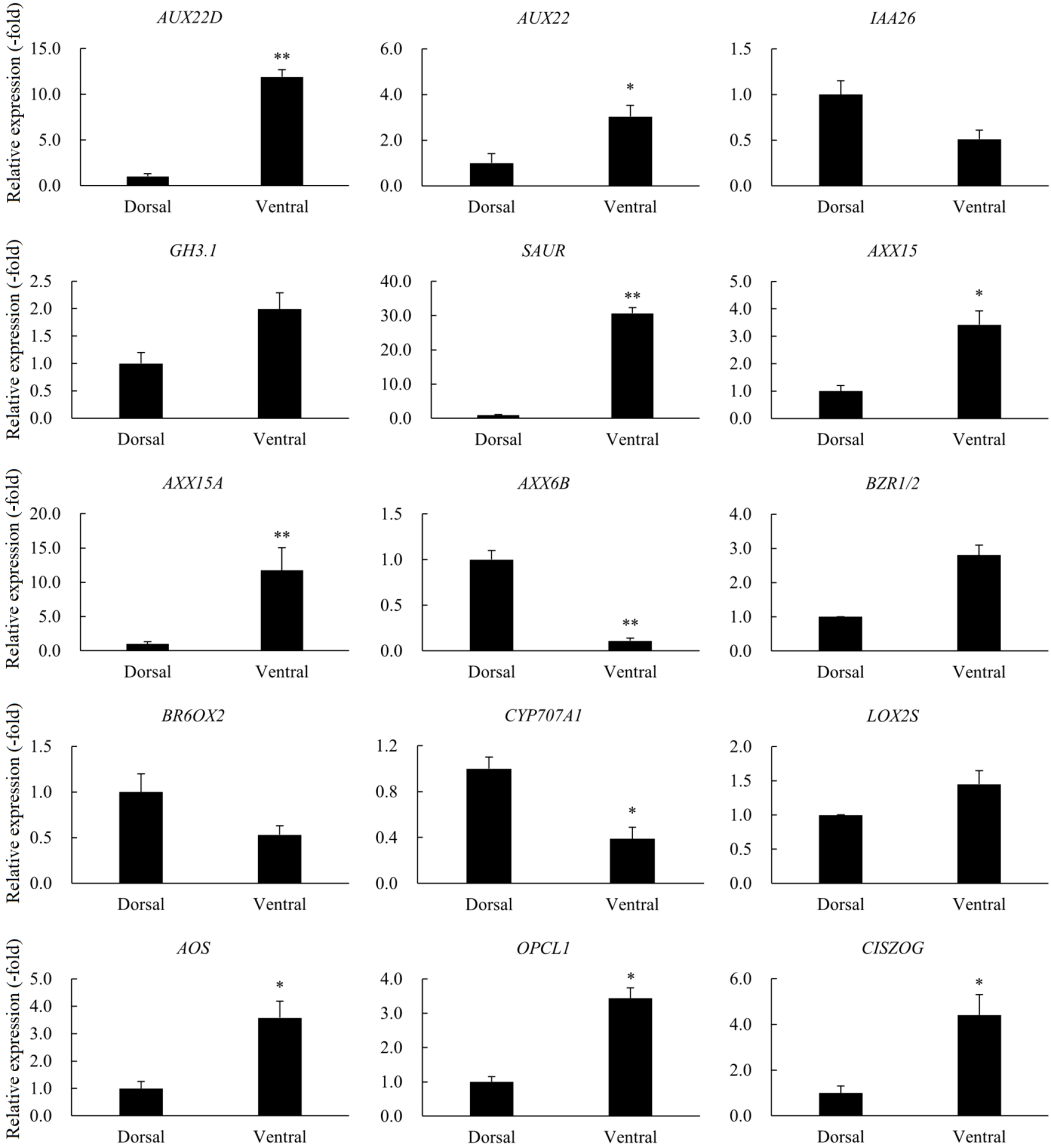
The relative expression of DEGs involved in hormone signaling in the dorsal and ventral stems of the ‘Blue bird’ water lily during stem bending, as determined by qRT-PCR. “*” and “**” represent significant difference level (*P*< 0.05 and *P<* 0.01) between the dorsal and ventral stems.

A total of 23 DEGs involved in hormone signaling in Dorsal_vs_Ventral were identified, including auxin, zeatin (ZT), JA, ABA and BR metabolic pathways genes (**Table 1**). RNA**-**seq showed that the relative expression of other genes was increased in the Dorsal_vs_Ventral except *IAA26*, *AXX6B*, *ARG7*, *AHP*, *BR6OX2*, *LUT5*, and *CYP707A1* (**Table 1**). qRT-PCR revealed that the trend in expression of 15 genes was consistent with the transcriptome data, of which *AUX22D*, *SAUR*, *AXX15A*, and *AXX6B* were highly significantly different in the Dorsal_vs_Ventral. *AUX22D*, *SAUR*, and *AXX15A* were increased 11.90-, 30.69-, 11.73-fold in the ventral stems, compared with the dorsal stems, respectively, and *AXX6B* was decreased 9.10-fold. *IAA26*, *GH3.1*, *BZR1/2*, *BR6OX2* and *LOX2S* were not significantly different, whereas all other genes were significantly different (**Table 1**, **Figure 8**).

**Table 1:** DEGs and RNA-seq expressions involved in hormone signaling in the dorsal and ventral stems of the water lily.

Gene name	Gene ID	FDR	Log_2_FC	up/down
*IAA31*	TRINITY_DN31826_c0_g1	0.000673	1.13	up
*AUX22D*	TRINITY_DN32832_c0_g1	0.001375	1.88	up
*IAA16*	TRINITY_DN34432_c0_g2	0.002277	1.28	up
*IAA17*	TRINITY_DN34432_c0_g4	0.003804	1.07	up
*AUX22*	TRINITY_DN38270_c1_g1	0.000469	2.05	up
*IAA1*	TRINITY_DN42468_c2_g1	2.09E-09	2.02	up
*IAA26*	TRINITY_DN40610_c0_g2	0.007946	−1.49	down
*GH3.1*	TRINITY_DN38483_c0_g4	0.000235	1.36	up
*SAUR*	TRINITY_DN30526_c0_g1	3.38E-06	4.12	up
*AXX15*	TRINITY_DN33080_c0_g1	0.001443	2.01	up
*AXX15A*	TRINITY_DN33931_c1_g2	0.007298	4.97	up
*AXX15A*	TRINITY_DN46640_c0_g4	0.000103	2.98	up
*AXX6B*	TRINITY_DN30678_c0_g1	3.01E-11	−5.03	down
*ARG7*	TRINITY_DN41116_c0_g1	0.004078	−1.77	down
*AHP*	TRINITY_DN43746_c1_g1	0.001355	−1.23	down
*BZR1/2*	TRINITY_DN46154_c0_g1	0.001924	1.14	up
*BR6OX2*	TRINITY_DN28579_c0_g1	0.000119	−1.38	down
*LUT5*	TRINITY_DN41861_c0_g2	3.17E-06	−1.44	down
*CYP707A1*	TRINITY_DN38908_c0_g1	0.000443	−1.43	down
*LOX2S*	TRINITY_DN43196_c0_g1	3.98E-07	2.38	up
*AOS*	TRINITY_DN30976_c0_g1	4.05E-05	1.92	up
*OPCL1*	TRINITY_DN41043_c0_g1	0.001989	1.53	up
*CISZOG*	TRINITY_DN43968_c0_g1	0.004797	1.81	up

### 3.5 DEGs involved in gravity, starch granules, and Ca^2+^ signaling

**Figure 9:**
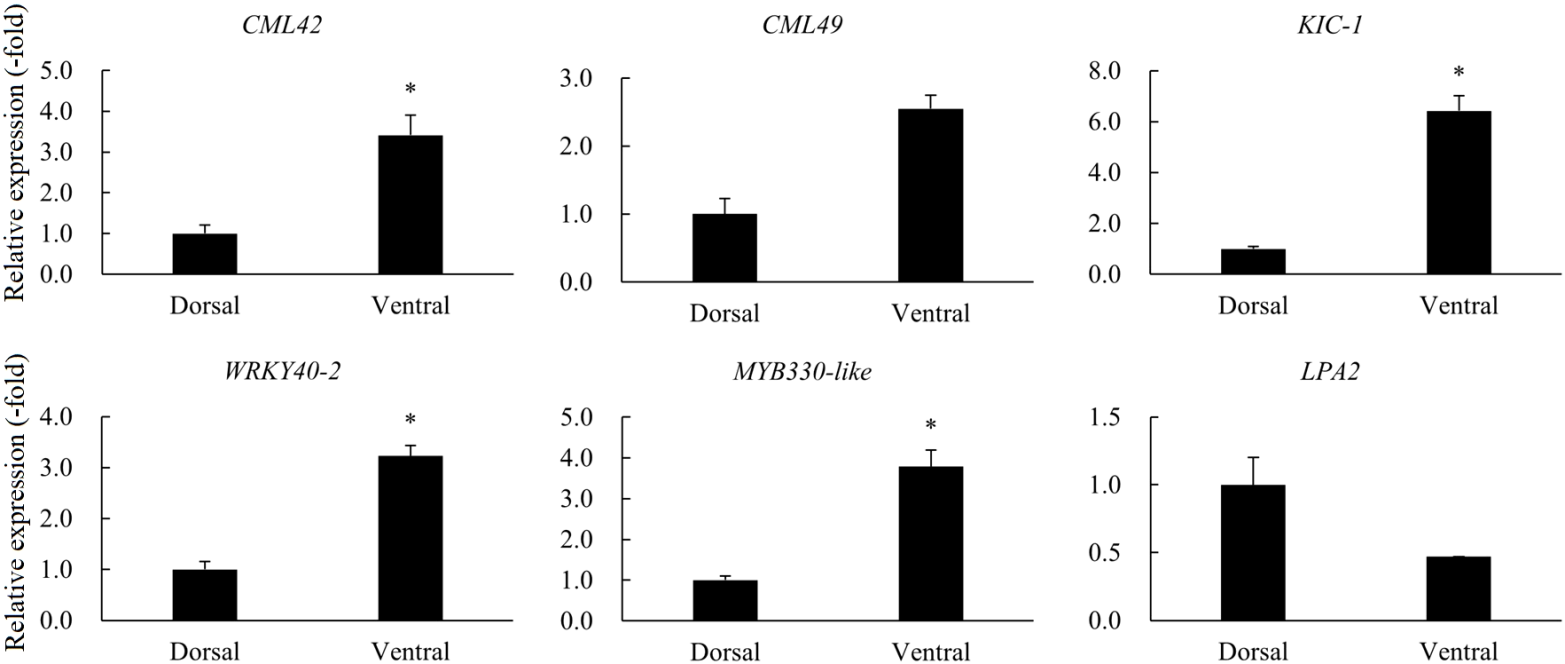
The relative expression of DEGs involved in gravity, starch granules, and Ca^2+^ signaling in the dorsal and ventral stems of the ‘Blue bird’ water lily during stem bending, as determined by qRT-PCR. “*” represents significant difference level (*P* < 0.05) between the dorsal and ventral stems.

A total of 12 DEGs were involved in gravity, starch granules, and Ca^2+^ signaling (**Table 2**). RNA-seq analysis revealed the DEGs *CML42* and *CML49*, which are associated with the major calcium receptor CaM-like protein (CML) in plants, were up-regulated in the ventral stems, compared with the dorsal stems. KIC, a calcium binding protein functioning in trichomes was also up-regulated. The transcription factors WRKY and MYB, which are associated with the synthesis of lignin and celluloses and thus affect the thickness of secondary cell walls and the mechanical strength of plant stems, were also significantly differentially expressed. Of these, the expression of the WRKY genes *WRKY75*, *WRKY18*, and *WRKY40* was up-regulated. The expression of the MYB genes *MYB44* and *MYB330-like* was also up-regulated. *LPA2* senses the gravity signals by affecting the synthesis and sedimentation of starch granules in plants. And RNA**-**seq showed that its expression was significantly down-regulated in the ventral stems, compared with the dorsal stems (**Table 2**). The expression of six other genes was consistent with the transcriptome data; *LPA2* was down-regulated 2.13-fold, and all other genes were up-regulated 2.55–6.42-fold as determined by qRT-PCR (**Table 2**, **Figure 9**).

**Table 2:** DEGs and RNA-seq expressions involved in gravity, starch granules, and Ca^2+^ signaling in the dorsal and ventral stems of the water lily.

Gene name	Gene ID	FDR	Log_2_FC	up/down
*CML42*	TRINITY_DN41755_c0_g3	0.00021	1.08	up
*CML49*	TRINITY_DN48382_c0_g1	0.000252	1.01	up
*KIC-1*	TRINITY_DN46389_c0_g1	0.000317	3.06	up
*KIC-2*	TRINITY_DN34835_c0_g1	1.11E-05	1.3	up
*WRKY75*	TRINITY_DN44747_c1_g1	0.008794	1.75	up
*WRKY18*	TRINITY_DN35911_c0_g2	0.000114	1.01	up
*WRKY40-1*	TRINITY_DN35911_c0_g1	0.002113	1.23	up
*WRKY40-2*	TRINITY_DN39344_c0_g1	7.75E-06	1.09	up
*MYB44-1*	TRINITY_DN40100_c0_g1	0.00654	2.2	up
*MYB44-2*	TRINITY_DN41812_c0_g1	9.57E-13	2.11	up
*MYB330-like*	TRINITY_DN31403_c6_g2	0.008376	1.72	up
*LPA2*	TRINITY_DN40026_c0_g1	4.84E-06	−1.38	down

### 3.6 DEGs involved in photosynthesis, peroxidase, and ABC transporters

**Figure 10:**
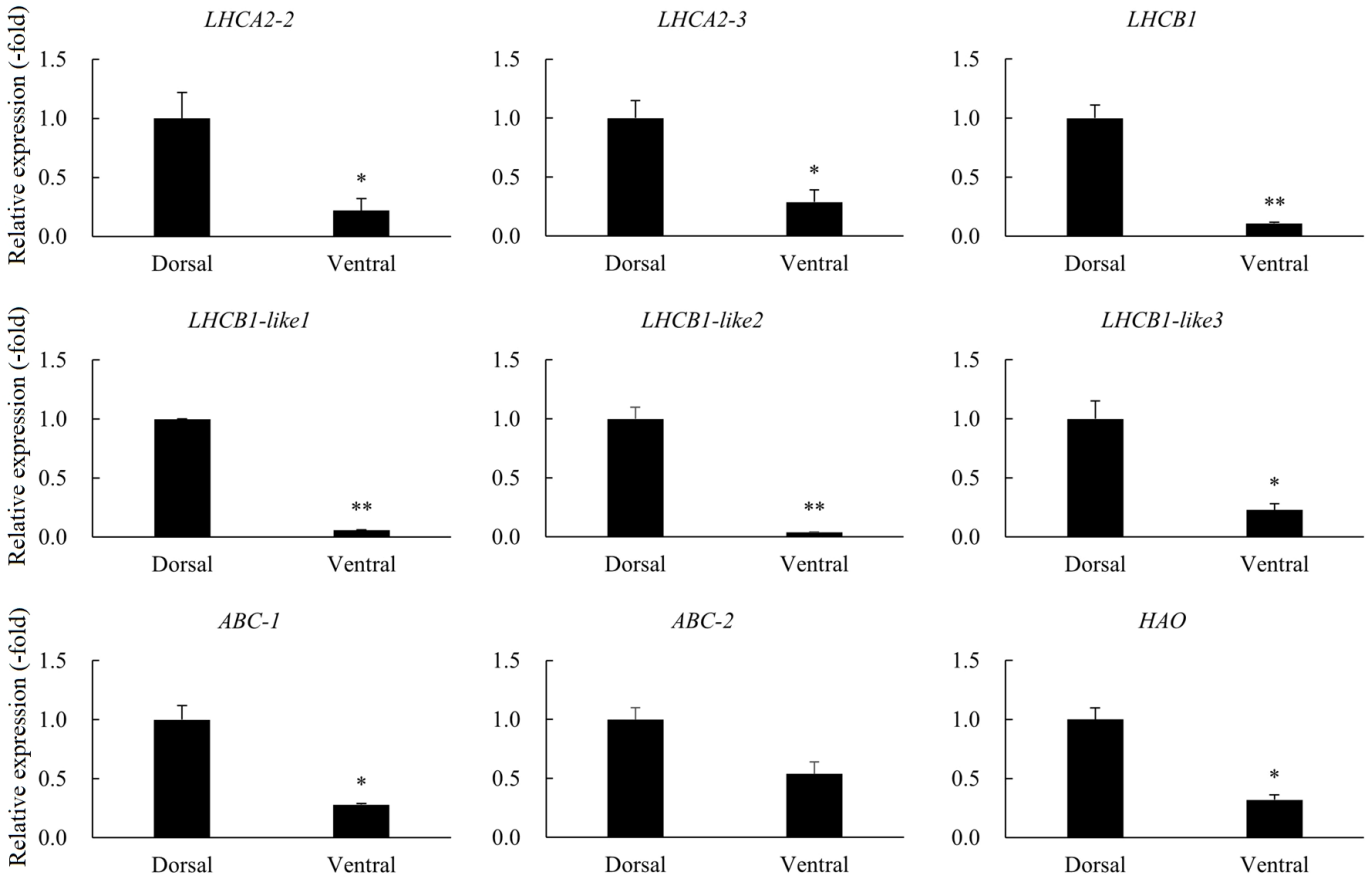
The relative expression of DEGs involved in photosynthesis, peroxidase, and ABC transporters in the dorsal and ventral stems of the ‘Blue bird’ water lily during stem bending, as determined by qRT-PCR. “*” and “**” represent significant difference level (*P*< 0.05 and *P<* 0.01) between the dorsal and ventral stems.

A total of 24 DEGs were involved in photosynthesis, peroxidase, and ABC transporters (**Table 3**). RNA-seq analysis revealed the expressions of *LHCA1*, *LHCA2*, *LHCA3*, *LHCA4*, *LHCA5*, *LHCB1*, *LHCB1-like*, *LHCB2*, *LHCB3*, *LHCB4*, *LHCB5*, *LHCB6*, *CAB1D*, *CAB1D-like*, and *CAB5* in pathways related to photosynthesis were significantly down-regulated in the ventral stems, compared with the dorsal stems. The expression of the peroxidase gene *HAO* and the ABC transporters genes were also down-regulated. The expression trend of nine other genes was consistent with that of the transcriptome data, and all genes were down-regulated 1.85–26.39-fold as determined by qRT-PCR (**Table 3**, **Figure 10**).

**Table 3:** DEGs and RNA**-**seq expressions involved in photosynthesis, peroxidase, and ABC transporters in the dorsal and ventral stems of the water lily.

Gene name	Gene ID	FDR	Log_2_FC	up/down
*LHCA1*	TRINITY_DN36720_c0_g1	1.41E-11	−1.86	down
*LHCA2-1*	TRINITY_DN33142_c0_g1	6.37E-23	−2.25	down
*LHCA2-2*	TRINITY_DN33142_c0_g2	5.37E-09	−2.37	down
*LHCA2-3*	TRINITY_DN37431_c1_g1	0.000147	−2.16	down
*LHCA3*	TRINITY_DN33438_c0_g1	0.000103	−1.46	down
*LHCA4*	TRINITY_DN38058_c1_g1	4.01E-08	−1.90	down
*LHCA5*	TRINITY_DN35522_c1_g2	0.000525	−2.12	down
*LHCB1*	TRINITY_DN34054_c0_g1	2.34E-05	−3.49	down
*LHCB1-like1*	TRINITY_DN38243_c1_g1	9.58E-05	−2.97	down
*LHCB1-like2*	TRINITY_DN39580_c0_g1	3.68E-06	−3.69	down
*LHCB1-like3*	TRINITY_DN49673_c1_g1	7.26E-07	−3.09	down
*LHCB1*	TRINITY_DN49673_c1_g2	3.30E-16	−2.07	down
*CAB1D*	TRINITY_DN49673_c1_g3	0.002611	−2.28	down
*CAB1D-like*	TRINITY_DN49673_c1_g7	8.01E-08	−2.46	down
*LHCB2*	TRINITY_DN38243_c1_g2	1.99E-05	−1.85	down
*CAB5*	TRINITY_DN49673_c1_g5	9.32E-09	−1.83	down
*LHCB3*	TRINITY_DN36703_c0_g1	0.000234	−2.24	down
*LHCB4*	TRINITY_DN31522_c0_g1	2.19E-05	−1.63	down
*LHCB5*	TRINITY_DN41916_c0_g1	3.84E-08	−1.82	down
*LHCB5*	TRINITY_DN41916_c0_g2	4.37E-15	−1.72	down
*LHCB6*	TRINITY_DN42125_c0_g2	3.99E-05	−1.80	down
*ABC-1*	TRINITY_DN33803_c1_g3	0.001748	−2.95	down
*ABC-2*	TRINITY_DN40514_c0_g1	1.77E-06	−1.11	down
*HAO*	TRINITY_DN44068_c0_g1	9.01E-05	−1.08	down

### 3.7 Changes in hormone concentrations in the dorsal and ventral stems

**Figure 11:**
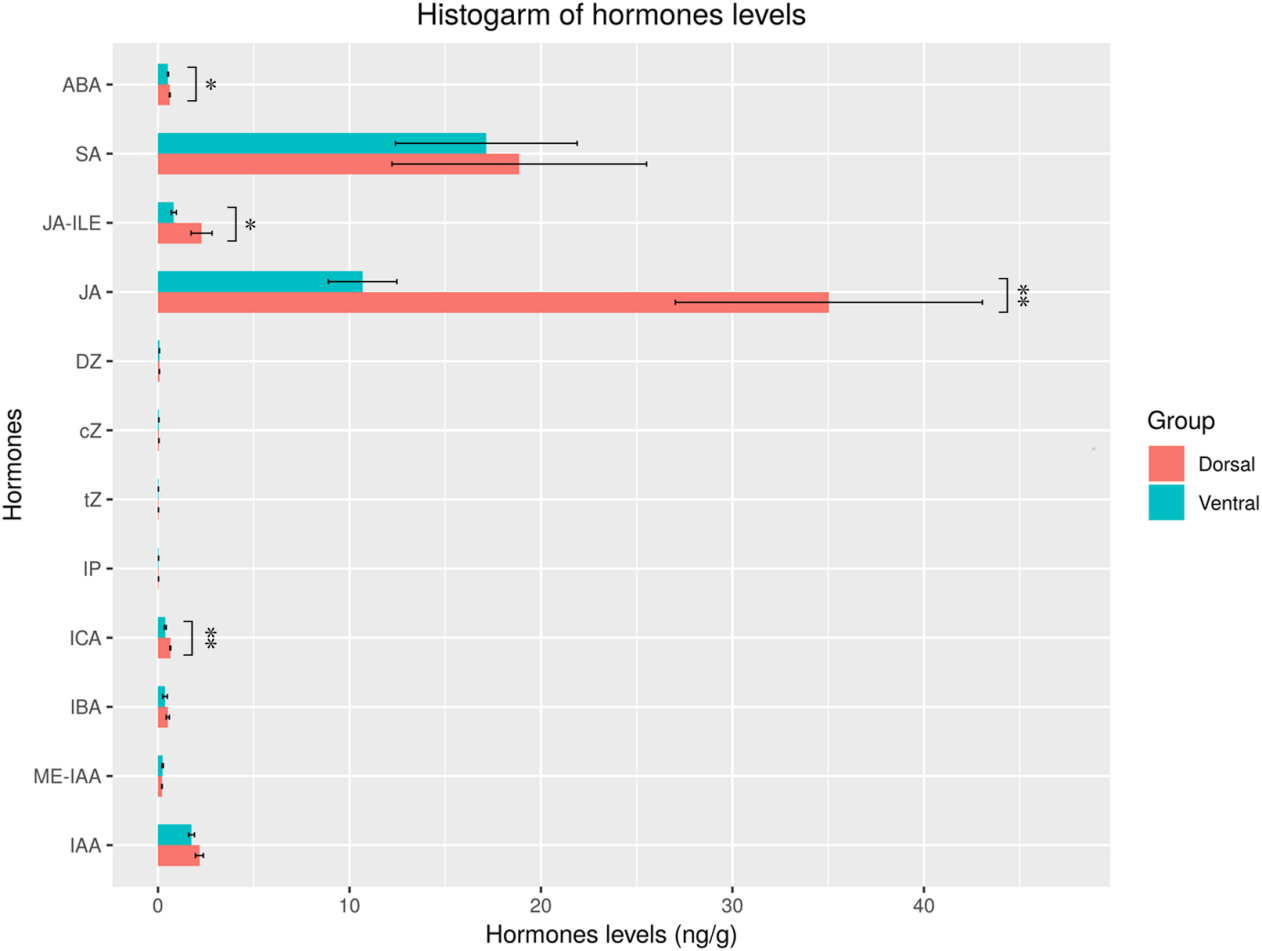
Hormone contents in the dorsal and ventral stems of the water lily. “*” and “**” represent significant difference level (*P*< 0.05 and *P<* 0.01) between the dorsal and ventral stems.

The levels of 15 hormones were measured in the dorsal and ventral areas of the ‘Blue bird’ water lily during stem bending, including indole-3-acetic acid (IAA), methyl indole-3-acetate (ME-IAA), 3-indolebutyric acid (IBA), indole-3-carboxaldehyde (ICA), N6-isopentenyladenine (IP), trans-zeatin (tZ), cis-zeatin (cZ), dihydrozeatin (DZ), methyl jasmonate (MEJA), JA, dihydrojasmonic acid (H2JA), jasmonoyl-L-isoleucine (JA-ILE), methylsalicylate (MESA), salicylic acid (SA) and ABA, using LC/MS/MS (**Table S6**, **Figure S4**). The results indicated that 12 hormones were detected, but 3 hormones (MEJA, H2JA, and MESA) were not detected (**Figure 11**). Of the hormones detected, the levels of ABA, SA, JA-ILE, ICA, IBA, ME-IAA, and IAA were relatively high. Moreover, the levels of ABA, ICA, JA-ILE, and JA were significantly different (*P*< 0.05) in the dorsal and ventral stems, with the levels in the dorsal stems being significantly higher than those in the ventral stems. There was a little difference in the levels of SA, cZ, tZ, DZ, IP, ME-IAA, IBA, and IAA between the dorsal and ventral stems (**Figure 11**).

## 4 Discussion

### 4.1 Effect of gravitropic response on stem bending

The gravitropic response of plants refers to a phenomenon in which plants perceive gravistimulation to relocate their own growth morphology and maintain the optimal angle of various organs in the direction of gravitropism (Firn and Digby, 1997; Perera et al., 2006; Song et al., 2021). Starch granule displacement and Ca^2+^ signals both affect the gravitropic response, because Ca^2+^, as an important second messenger, is involved in various steps of the response (Aarrouf and Perbal, 1996; Philosoph-Hadas et al., 1996; Sinclair and Trewavas, 1997; Toyota et al., 2008; Zhang et al., 2011). In this study, the starch granules settlement in pedicel cells of water lily was consistent with the bending direction of the water lily stem, as well as the direction of gravity (**Figures 4**, **5**), indicating the gravitropic response is an important cause of the stem bending of water lily cut-flowers.

Studies have shown that starch granules in endodermal cells stimulated by gravity precipitate along the direction of the stimulation. The *Loose Plant Architecture 1* (*LPA1*) gene can sense gravity signals by regulating the synthesis and sedimentation of starch granules in plants. *LPA1* mutation affects the perception of gravistimulation, resulting in a loose shoot architecture in rice, which is in contrast to the rather compact shoot system of the wild-type plant (Wu et al., 2013). Secondary signaling molecules, such as Ca^2+^, are activated, which transform gravity signals into physiological and biochemical signals (Wu et al., 2013; Wu et al., 2016; Jiao et al., 2021). Kinesin-like calmodulin binding protein (KCBP) is a microtubule motor protein involved in the regulation of cell division and trichome morphogenesis. Reddy et al. (2004) have shown that calcium can bind to KCBP-interacting Ca^2+^ binding protein (KIC), then interact with KCBP and inhibits KCBP microtubule binding activity and microtubule stimulated ATPase activity. Poovaiah et al. (2013) suggested that calmodulin (CaM) and CaM-like protein (CML) are the primary calcium receptors in plants. The study found intracellular calcium receptor perception of changes in intracellular calcium ion concentration, in response to the NAC, MYB (such as MYB330), and WRKY (such asWRKY40) transcription factors, which affected the biosynthesis of the secondary cell wall, resulting in changes in lignin and cellulose accumulation. This affects the thickness of the secondary cell wall and attenuates stem mechanical strength (Tang et al., 2019). Lear et al. (2022) used RNA-seq to analyze gene expression at three stages of pedicel necking in *Rosa hybrida* (straight,<90° and >90°) and found that more genes down-regulation than up-regulation during necking; moreover, most NAC and WRKY transcription factors involved in stress and senescence were up-regulated.

In this study, the calcium signal-related genes *CML* and *KIC* as well as some WRKY and MYB transcription factors related to the calcium signal-related genes were up-regulated in the ‘Blue bird’ water lily during stem bending (**Figure 9**). These results are in general agreement with those of Lear et al. (2022). At the same time the *LPA2*, which senses the effect of gravity by affecting the settling of starch granules in the dorsal and ventral stems, was down-regulated. These results indicated the starch granules settlement to the ventral parts of the cells activated the calcium signal-related genes and transcription factors, which resulted in an uneven distribution of auxin in the plant, and finally induced a curved growth of stem of water lily.

On the other hand, during the vase life, because of the heavy head of the water lily flower, the stem is squeezed against the bottle wall, resulting in different squeezing forces on the dorsal and ventral areas. This affects the growth and arrangement density of the cells in the dorsal and ventral areas during stem bending, which results in a change in the mechanical force on the stem. Longitudinal paraffin sections prepared from bent stems showed no significant difference in the cell morphology of the dorsal and ventral areas on the 1st day. However, on the 5th day, the cell size and arrangement density in the dorsal and ventral areas were different (**Figure 2**). Thus, the change of cell morphology by gravity may also another cause of stem bending of water lily flower stem.

### 4.2 Effect of hormone signaling on stem bending

Plants perceive stimulatory signals and respond rapidly by remodeling intracellular processes and rebalancing the utilization of limited resources between growth and defense responses (Ning et al., 2017; Ye et al., 2018; Lakehal et al., 2019). Among them, phytohormones act as mediators that sense gravity. They are transported between tissues and accumulate in target tissues, causing an asymmetric distribution of phytohormones (Philosoph-Hadas et al., 2005). The Cholodny-Went model suggests that auxin is the primary mediator of gravitropism (Went and Thimann, 1937). In this study, as for the auxin, the content of ICA was significantly higher (*P*< 0.05) in the dorsal part than that of ventral part, while IBA, ME-IAA, and IAA were not significantly higher in the dorsal part, which could explain the pedicel bending because the dorsal part grew more quickly than the ventral part (**Figure 11**).

Studies have shown that other hormones act synergistically with auxin to participate in the gravitropic bending process (Wang et al., 2015). ABA, JA, GA_3_, BR and CK have all been demonstrated to participate in the regulation of plant gravity response. The interactions among these hormones determine the overall response of the stem to external stimuli or internal signals, regulating the growth and development processes that influence stem bending. Study has shown that the IAA and ABA amounts were larger on the tension wood side than on the opposite wood side and in upright trees after 3 weeks of bending, and found that the distribution patterns of IAA and ABA might have important roles in tension wood formation (Kijidani et al., 2023). ABA can weaken the root gravitropic response under conditions of water stress (Taniguchi et al., 2010). MeJA was found to be a key phytohormone for determining the branching angle of *Arabidopsis* lateral roots, which is dependent on canonical JAR1-COI1-MYC2,3,4 signaling to incline lateral roots to a more vertical orientation. JA can synergistically interact with auxin, and light signals were found to enhance JA biosynthesis leading to the erect root architecture, whereas glucose induced wider branching angles (Sharma et al., 2022). GA_3_ is typically involved in cell elongation in conjunction with IAA. Muday’s research has shown that after gravity treatment, gibberellins exhibit uneven distribution on both sides of the stem reaction site, participating in the regulation of uneven growth along with auxin (Muday, 2001). BR biosynthesis and signal transduction play important roles in regulating plant agronomic traits, including plant height, leaf angle, grain size, and flowering (Tong and Chu, 2018; Nolan et al., 2020). BR can induce high expression of *PIN2*, facilitating polar auxin transport to establish concentration gradients, thereby accelerating the gravity response (Kim et al., 2000; Li et al., 2005). High concentrations of BR treatment inhibit plant gravity response, whereas low concentrations of BR can promote gravity response (Vandenbussche et al., 2011). Tian et al. (2021) found that the rice bHLH transcription factor OsBIM1 functions as a positive regulator in BR signaling and its overexpression significantly increases rice leaf angles by enhancing BR sensitivity and response. Low-concentration ABA increases leaf inclination in rice through a synergistic effect by inducing the expression of the BR-biosynthesis regulatory gene *OsGSR1*, to activate the BR signal in a fast, limited and short-term manner (Li et al., 2021). CKs are phytohormones involved in shaping rice architecture. Huang et al. (2023) used a phenotypic analysis to show that the leaf angle of a rice *OCSKX3* mutant was smaller, whereas the leaf angle of overexpressed lines (*OcSKX3-OE*) was larger. Histological sections showed that the changes were caused by an asymmetric proliferation of ganglion cells and vascular bundles, which indicates that enhancing CK levels in the lamina joint by disrupting *OsCKX3* negatively regulates leaf angle. In this study, in all phytohormones, the levels of ABA, JA-ILE, and JA were significantly different (*P*< 0.05) in the dorsal and ventral stems, suggesting that ABA and JA exert certain effects on water lily stem bending, but the specific mechanism still needs to be elucidated.

### 4.3 Expression of hormone-related DEGs on stem bending

Ge et al. (2019) found that the expression of many unigenes involving in signaling of phytohormones, such as auxin, CTK, GA, ABA, ethylene, BR, and SA showed significantly changes during the stem bending process of gerbera. In our study, 23 DEGs involved in hormone signaling in Dorsal_vs_Ventral were identified, including auxin, zeatin (ZT), JA, ABA and BR metabolic pathways genes (**Table 1**). *IAA26*, *AXX6B*, *ARG7*, *AHP*, *BR6OX2*, *LUT5*, and *CYP707A1* were down-regulated, and the other DEGs were up-regulated (**Table 1**). As for the auxins, previous studies have indicated that changes in auxin biosynthesis, transportation, and signal transduction lead to abnormal gravity, and finally changes in the branch angle (Roychoudhry et al., 2013; Roychoudhry and Kepinski, 2015; Zhang et al., 2018; Li et al., 2019; Ke et al., 2021; Zhu et al., 2021). Our study revealed differential expression of auxin-related genes in the dorsal and ventral parts of the water lily stem, such as the Aux/IAA proteins are short-lived transcription factors that repress early auxin response genes at low auxin concentrations (Liscum and Reed, 2002). *GH3.1* encodes IAA-amido synthetases, which help to maintain auxin homeostasis by conjugating excess IAA with amino acids (Staswick et al., 2005). Our transcriptome and qRT-PCR studies revealed that these genes presented significantly expression difference between the dorsal part and the ventral part, which were consistent with hormone contents (**Table 1**, **Figure 8**). Thus, we speculate that these differentially expressed genes may regulate stem bending in the water lily by regulating the expression of auxin, but further verification is needed for their specific functions.

Moreover, our study revealed that other plant hormones also exhibited differential expression between the dorsal and ventral parts of the water lily stem. For example, as for the BR-related DEGs, *BZR1* coordinates BR homeostasis and signaling by playing dual roles in mediating the downstream growth response and negative feedback regulation of BR biosynthesis (He et al., 2005). *BR6OX2* catalyzes the C6-oxidation step and lactonization during BR biosynthesis (Shimada et al., 2001). *LUT5* and *CYP707A1* are involved in carotenoid biosynthesis and ABA catabolism (Kim and Dellapenna, 2006; Saito et al., 2004). *LOX2S*, *AOS*, and *OPCL1* are involved in the biosynthesis of JA, thereby regulating a wide variety of growth development and defense-related processes (Royo et al., 1996; Koo et al., 2006). *CISZOG* is involved in the CK metabolic process and encodes zeatin o-xylosyltransferase, which is considered important for storage and protection against degradative enzymes (Martin et al., 1999). qRT-PCR validation was consistent with the transcriptome results, leading us to propose that these differentially expressed genes may play important roles in stem bending in the water lily by participating in the metabolism of plant hormones.

### 4.4 Effect of light on stem bending

The plant branching angle is regulated by several factors, including gravity stimulation, phytohormones, and the external environment, such as light and water (Bai et al., 2013; Roychoudhry et al., 2017; Sharma et al., 2022). The light-harvesting complex (LHC), as a type of photoreceptor, is involved in regulating plant growth and development (Wientjes et al., 2011). Light serves as a critical environmental signal, playing a significant role in shaping plant architecture. Our study revealed that the expressions of 15 DEGs (*LHCA1*, *LHCA2*, *LHCA3*, *LHCA4*, *LHCA5*, *LHCB1*, *LHCB1-like*, *LHCB2*, *LHCB3*, *LHCB4*, *LHCB5*, *LHCB6*, *CAB1D*, *CAB1D-like*, and *CAB5*) related to photosynthesis were significantly down-regulated in the ventral stems, compared with the dorsal stems (**Table 3**). Extensive research has shown that under different light conditions, plant growth direction and branching angles are induced through different pathways to varying degrees. For instance, studies have demonstrated that the *Tillering Angle control 1* (*TAC1*) plays a role in the lateral branch orientation of different plant species. Waite and Dardick (2018) found that *TAC1* expression is light-dependent, and *TAC1* overexpression partially prevents the reduction of the branch angle under dark or far-red light conditions. In addition, studies have found that changes in *TAC1* in other plant species are also related to upright tillers or branch angles (Jiang et al., 2012; Dardick et al., 2013; Hollender et al., 2018). Rice *OsPIL15* integrates light and gravity signals to negatively regulate the tiller angle in rice (Xie et al., 2019). Furthermore, a study found that ABC transporters function in the auxin transport phase of gravitropism (Strohm et al., 2012). Another study showed that ABC transporters (*abcb19*) hypocotyls respond to gravistimulation twice as quickly as wild-type plants, and they also exhibit an enhanced phototropic response (Noh et al., 2003).

Water lilies are very sensitive to light. During vase life, the pedicel ventral is close to the bottle wall, so the light intensity at the ventral area is lower than that at the dorsal area. This difference in light may also affect the growth of the dorsal area. In this study, many genes related to photosynthesis, carbon metabolism, and photosynthesis-antenna proteins were identified (**Figures 7B**, **10**). The expression levels of photosynthesis-related genes in the dorsal area were significantly higher than those in the ventral area; these findings were consistent with those of a study by Lear et al. (2022), who found that photosynthetic, starch, and lignin biosynthesis genes were all down-regulated during pedicel necking in *Rosa hybrida*. This indicated that light response and photosynthesis in the pedicel ventral were significantly lower than those in the dorsal area due to weak light exposure. As a result, more photosynthetic products accumulate in the dorsal area, resulting in relatively stronger dorsal growth and stem bending. In summary, although in this study the differential expression of photosynthesis, peroxidase, and ABC transporters related DEGs in the dorsal and ventral area suggested they may play important roles in the development of water lily stems bending, the specific regulatory mechanism is still unclear, and the further verification of the functions of these genes should be carried out in the future.

### 4.5 Speculated pathways of DEGs regulating stem bending

**Figure 12:**
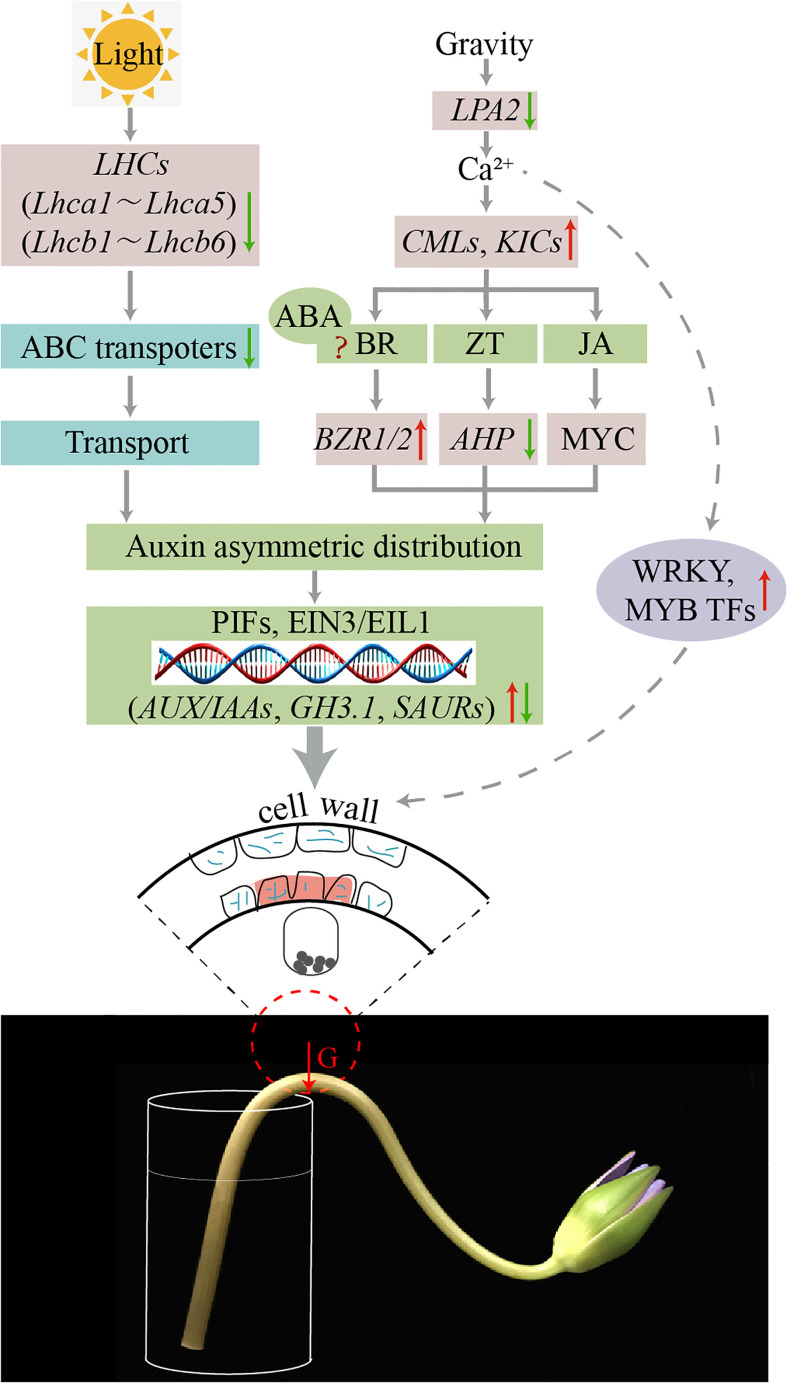
Schematic representation of putative pathways of DEGs that regulate stem bending in the ‘Blue bird’ water lily.

Cai (2014); Kruse and Wyatt (2022) summarized the possible pathways of the direction of tropic movement relating to the direction of the stimulus, which can be induced by gravity, light, moisture, and chemicals. In this study, according to the results of stem bending at the transcriptional, metabolic and cellular level in water lily cut-flowers, we hypothesized that gravity, light and the uneven distribution of hormones affects cell growth in the dorsal and ventral parts, which causes stem bending (**Figure 12**). At the transcriptional level, genes related to plant hormone synthesis pathways, gravity, starch granules, Ca^2+^ signaling, and photosynthesis were differentially expressed in the dorsal and ventral parts of the bent water lily stems. Gene expression measured by qRT-PCR was consistent with the transcriptome sequencing results. At the metabolic level, significant differences in hormone content were observed between the dorsal and ventral areas, and ABA, ICA, JA-ILE and JA were significantly levels. At the cellular level, during vase life, the water lily stem was significantly bent, the cell morphology of the dorsal and ventral areas was changed, and starch granules in the bent stem cells were precipitated in the same direction as the bent stem and the direction of gravity. In conclusion, during vase life of water lily cut-flowers, the deposition of starch granules in the bent stem cells resulted in asymmetric auxin distribution and a change in the secondary cell wall, which affected the mechanical strength of the stem. Moreover, the higher weight of the water lily flowers head results in different forces on the dorsal and ventral areas, resulting in changes in cell size and arrangement density and affecting the mechanical strength of the stem. Uneven light exposure to the dorsal and ventral areas resulted in the downregulation of gene expression related to photosynthesis, differences in the accumulation of photosynthetic products, and growth rate, which contributed to stem bending.

## 5 Conclusion

In this study, the DEGs identified in different areas of the water lily cut-flowers suggest transcription-based regulation of stem bending. This comparative transcriptome analysis focused on the pathways mediating the occurrence of cut-flowers stem bending including hormone signaling, gravity response, and light pathways. This was confirmed by the measurement of hormone levels in the stem and dynamic changes in cell morphology and starch granules. According to the results, the main possible causes of the bending of water lily cut-flower stems are the higher concentration auxin of ICA on the dorsal part to the ventral part due to gravity and light. In this progress, calcium signaling may play an important role as the second messenger. Therefore, auxin transport inhibitors and calcium channel blockers could be used to treat the cut-flowers, reducing the asymmetric distribution of auxin and relieving the bending phenomenon. At the same time, ABA, MeJA and JA also had the higher concentrations on the dorsal part compared to the ventral part, so the antagonistic substances of these hormones such as gibberellin may also alleviate the bending phenomenon.

The results of this study can provide ideas for subsequent treatment of water lily cut-flowers with various fresh-keeping methods, but what kind of exogenous hormones can effectively prolong the vase life of water lily cut-flowers needs further research and will be the focus of our next research. Meanwhile, although the genes associated with stem bending have been identified and mapped in this study, the role of these genes in regulating stem bending needs to be elucidated in future studies. Ideally, it would be desirable to develop varieties less prone to stem bending through molecular breeding. Therefore, we expect that the findings of this study will be valuable for prolonging the occurrence of cut-flowers that experience early stem bending issues.

## Data availability statement

The original contributions presented in the study are publicly available. This data can be found here: https://www.ncbi.nlm.nih.gov/bioproject/PRJNA934415 (to be released on acceptance).

## Author contributions

JL, YS, YaZ, YiZ, XS, and JW participated in the experimental design and data analysis. JL, YS, HX, QL, and XL performed the experiments. JL, YS, and JW wrote the paper. All authors contributed to the article and approved the submitted version. JL and YS contributed equally to this paper.
