# Supplementary material for: Transcriptome and hormone metabolome reveal the mechanism of stem bending in water lily (Nymphaea tetragona) cut-flowers
Source: Front Plant Sci. 2023 Sep 8;14:1195389. doi: 10.3389/fpls.2023.1195389 (PMC10515221; doi:10.3389/fpls.2023.1195389)
Supplement: Supplementary file 1 [file DataSheet_1.docx]

***Supplementary Materials***

**Transcriptome and hormone metabolome reveal the mechanism of stem bending in water lily (*Nymphaea tetragona*) cut-flowers**

**Jie Li^1†^, Yuhui Sheng^1†^, Tingge Li^1^, Tongxin Wang^1^, Qinxue Li^1^, Xiuya Lin^1^, Yang Zhou^1^, Ying Zhao^1^, Xiqiang Song^1^, Jian Wang^1*^**

**^*^Corresponding authors**: Jian Wang, e-mail: wjhainu@hainanu.edu.cn

## Supplementary Figures

**
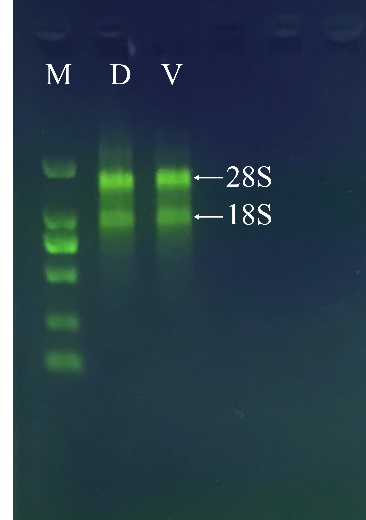
**

**Figure S1.** Detection results of RNA of dorsal and ventral stems by agarose gel electrophoresis. M: marker, D: dorsal stem, V: ventral stem.


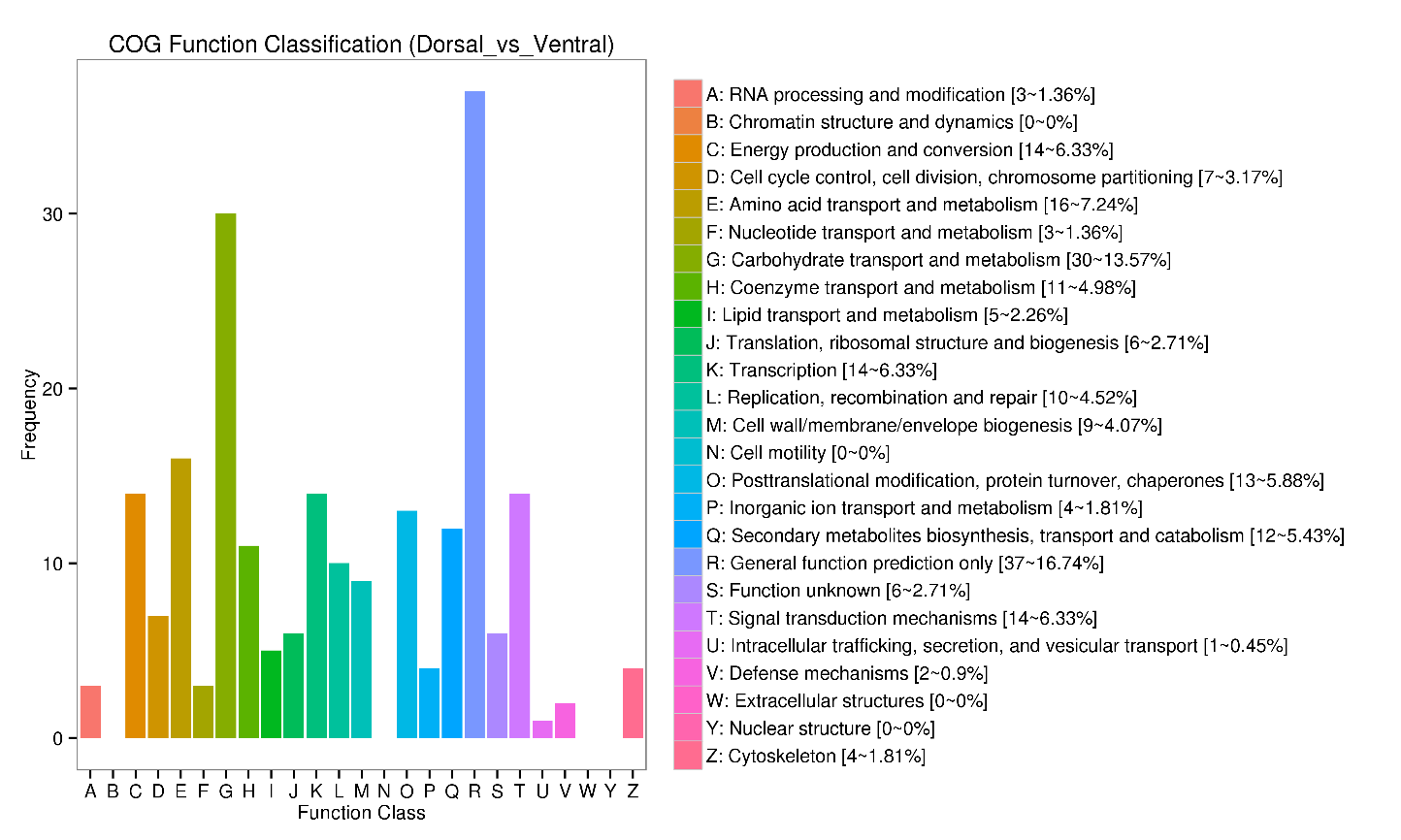


**Figure S2.** COG annotation classification of DEGs.


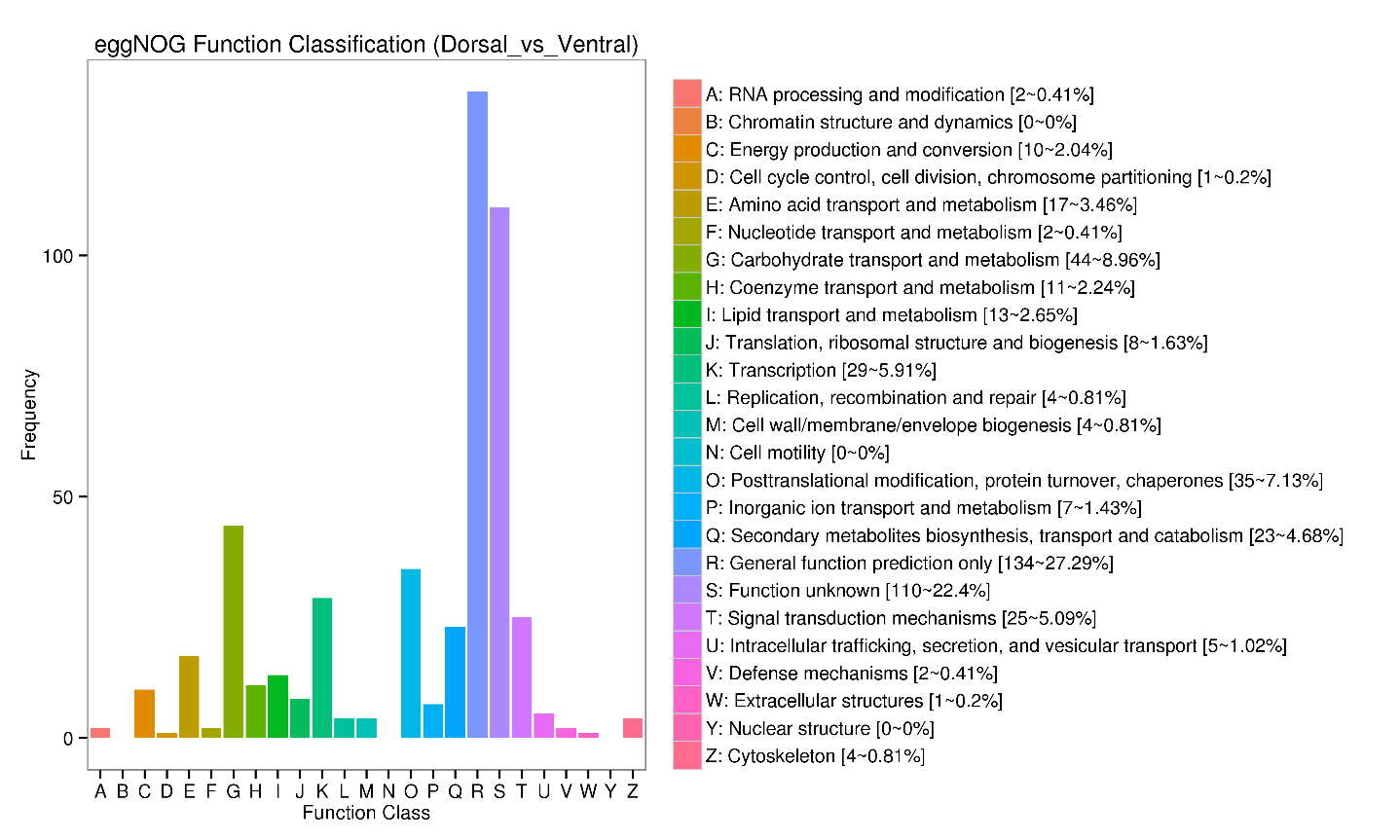


**Figure S3.** eggNOG annotation classification of DEGs.


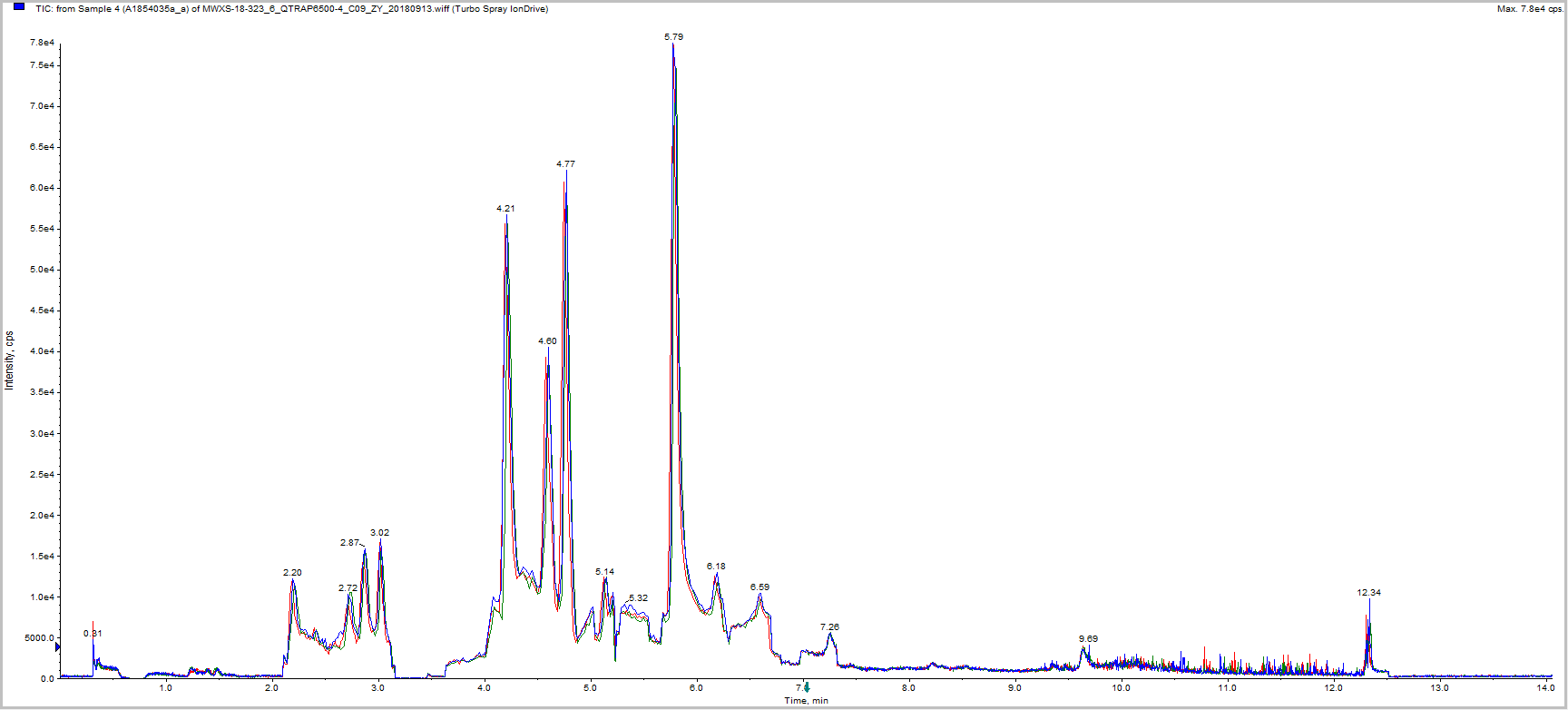


**Figure S4.** TIC overlap diagram.

# Supplementary Tables

**Table S1.** Primer used for qPCR.

| **Genes** | **Sequences (5' to 3')** |
| --- | --- |
| Actin-F | GAAGCCCAGTCCAAAAGAGGT |
| Actin-R | TGGCATAAAGAGAGAGCACGG |
| TRINITY_DN38270-c1-g1-F | GAACGAGGAAGTGGTGGAGGA |
| TRINITY_DN38270-c1-g1-R | GTGGCTCTTGAGGTGGATTTT |
| TRINITY_DN40610-c0-g2-F | AGAGTGAAAGGGCAGGGGATT |
| TRINITY_DN40610-c0-g2-R | ATAATCGGCTTCTCTCCTCAG |
| TRINITY_DN38483-c0-g4-F | AGGGTGCTGGCGGAGATACTG |
| TRINITY_DN38483-c0-g4-R | AGATGGGGTGAGCAGATAGGA |
| TRINITY_DN33080-c0-g1-F | ATGCTGAGGCAATGGCGAAAG |
| TRINITY_DN33080-c0-g1-R | CGTGGTCGTAGCCGAACTCCT |
| TRINITY_DN33931-c1-g2-F | CCTGCCTCTGTCAGTTCTTGC |
| TRINITY_DN33931-c1-g2-R | CTGTGATGCTCAAAACCGAAC |
| TRINITY_DN43746-c1-g1-F | AGCCAGGGGTTTGGTGTAGTA |
| TRINITY_DN43746-c1-g1-R | ACTGCCCATCAAGATAACCCT |
| TRINITY_DN46154-c0-g1-F | ACAATGAAGCGGAAAGCGGTA |
| TRINITY_DN46154-c0-g1-R | TGTTTCCCAAGGACCAATCGC |
| TRINITY_DN33142-c0-g2-F | GCGTTCTCAACACCCCTTCAT |
| TRINITY_DN33142-c0-g2-R | TTCTACCCTCTGCCCACCCTA |
| TRINITY_DN37431-c1-g1-F | AGGGGGTCTCTGCCGTATGTG |
| TRINITY_DN37431-c1-g1-R | GCACTTCTGGGACAAGGATTC |
| TRINITY_DN34054-c0-g1-F | TGGACCGCTGGGTGAAGTGAC |
| TRINITY_DN34054-c0-g1-R | CAGGGACGAAGTTGGTGGCAT |
| TRINITY_DN38243-c1-g1-F | CCTCCTCTGCCTCCAACCTGC |
| TRINITY_DN38243-c1-g1-R | CAGCGGTGTCCCAGCCGTAGT |
| TRINITY_DN49673-c1-g1-F | CTCTGGTAGCCCTTGGTATGG |
| TRINITY_DN49673-c1-g1-R | AACCACACAGACTCCCCGAAT |
| TRINITY_DN32832-c0-g1-F | \| CTCCACAGTCGTCCCCTAAGA \| \| --- \| |
| TRINITY_DN32832-c0-g1-R | CATCCTGTAAGAGCAGACGGG |
| TRINITY_DN30526-c0-g1-F | TTGGAATCTCGCCACCTGAAT |
| TRINITY_DN30526-c0-g1-R | CCGAGTGGCAGAAAACTTGGT |
| TRINITY_DN30678-c0-g1-b-F | AGTGGAGAAATGAAGGAGGGC |
| TRINITY_DN30678-c0-g1-b-R | TCCAATCCCCTGCCAATAGTC |
| TRINITY_DN39580-c0-g1--a-F | TCTCACTCAACCCACTCATCG |
| TRINITY_DN39580-c0-g1-a-R | GCTACTGGAGGAGACAGGCTT |
| TRINITY_DN41755_c0_g3-F | TCCGCAATCCCAGCCTCAACA |
| TRINITY_DN41755_c0_g3-R | CCCCGCTTCCAAAGAACCCCT |
| TRINITY_DN34835_c0_g1-F | GCAGATGGTGAGGGAAGGAGA |
| TRINITY_DN34835_c0_g1-R | ATCTTCTCATCAACTCGGGGC |
| TRINITY_DN48382_c0_g1-F | ATCGCACTCCAGCATCTAAAT |
| TRINITY_DN48382_c0_g1-R | CGTTGCTGTTCATCAATCCAT |
| TRINITY_DN46389_c0_g1-F | ACGGCTTCAGGATGCTTATGG |
| TRINITY_DN46389_c0_g1-R | CATCAACCCTGGGCTCAATCT |
| TRINITY_DN39344_c0_g1-F | CTCTTGTGCTGTTGGTCTGTC |
| TRINITY_DN39344_c0_g1-R | AAGTGGGTATGGTTTTCCTGT |
| TRINITY_DN31403_c6_g2-F | GGGTAGAAGTCCTTGCTGCTC |
| TRINITY_DN31403_c6_g2-R | TTCCTCTCTTGATGTCGGGTC |
| TRINITY_DN40026_c0_g1-F | CGATGCCTCCGTTCTTTCACA |
| TRINITY_DN40026_c0_g1-R | CGACCTTCCTCTTCCTGCTGC |
| TRINITY_DN33803_c1_g3-F | TCTCTCAGTGAATGCCAAATG |
| TRINITY_DN33803_c1_g3-R | TTATCTAATCTTCCCGCCAGT |
| TRINITY_DN40514_c0_g1-F | GCGGCAGTCTCGTGCTTTCTA |
| TRINITY_DN40514_c0_g1-R | TCTCTCTGTTTCCAGGGCTCT |
| TRINITY_DN28579_c0_g1-F | AAATCTGCCAGGAACCAACTA |
| TRINITY_DN28579_c0_g1-R | GTATCCAACAAGATTCCAAGC |
| TRINITY_DN41861_c0_g2-F | CAACTGGGGCAACCATTCATA |
| TRINITY_DN41861_c0_g2-R | GCAGGTTTATCTGGACGAGGC |
| TRINITY_DN38908_c0_g1-F | TCCCAAAATCCCAATGTCTTC |
| TRINITY_DN38908_c0_g1-R | TGGCTCTGACGACCAACTTCC |
| TRINITY_DN43196_c0_g1-F | CCTTGAAACCCATTGCCATCG |
| TRINITY_DN43196_c0_g1-R | CCAGTATGAACGGCTCCACGC |
| TRINITY_DN30976_c0_g1-F | GACCAGCACACCAGGCTCAAG |
| TRINITY_DN30976_c0_g1-R | ATTTCAAGCAAAGGCTCGCAC |
| TRINITY_DN41043_c0_g1-F | CAAGGCGTGAACCCGAGAAGT |
| TRINITY_DN41043_c0_g1-R | CGATGGGGAAGTGGATGGAGT |
| TRINITY_DN43968_c0_g1-F | ATGTGCCGTGTGAGAGATGCG |
| TRINITY_DN43968_c0_g1-R | GGAGATGGAGGGGAAGGAAGC |
| TRINITY_DN44068_c0_g1-F | GCTGCCTGGAAGAGGTTGTTA |
| TRINITY_DN44068_c0_g1-R | AAACACCAGAAGCACCAAGCG |
| TRINITY_DN47071_c0_g1-F | ATACAGAAGGTCCATACAAGC |
| TRINITY_DN47071_c0_g1-R | ATTCTCCTCTCCAACAATCTT |
| TRINITY_DN31006_c0_g1-F | GAGAAAATGGATGAGGTTGAT |
| TRINITY_DN31006_c0_g1-R | AGAAGTGACGCTCTCAATACG |
| TRINITY_DN38352_c1_g1-F | CTCCCTCCGTTTCCTGCTTAT |
| TRINITY_DN38352_c1_g1-R | TCGGTTGTTGAACTCGTGGCT |

**Table S2.** Quality control data of transcriptome sequencing.

| **Sample** | **Clean reads** | **Clean bases** | **Q20** | **Q30** | **GC content** |
| --- | --- | --- | --- | --- | --- |
| Dorsal1 | 46,196,006 | 6,929,400,900 | 97.94 | 94.74 | 48.20 |
| Dorsal2 | 46,989,812 | 7,048,471,800 | 98.06 | 95.00 | 48.16 |
| Dorsal3 | 46,528,376 | 6,979,256,400 | 98.02 | 94.88 | 48.46 |
| Ventral1 | 46,576,648 | 6,986,497,200 | 98.15 | 95.20 | 48.18 |
| Ventral2 | 46,695,622 | 7,004,343,300 | 97.77 | 94.28 | 48.55 |
| Ventral3 | 40,700,410 | 6,105,061,500 | 97.80 | 94.23 | 48.26 |

Q20, the percentage ratio of the base number and total base number with Qphred no less than 20;

Q30, the percentage ratio of the base number and total base number with Qphred no less than 30;

GC content, the percentage ratio of the G and C content and the total base number in clean reads.

**Table S3.** Reads and reference genome mapping data.

| **Sample** | **Mapped Reads** | **Mapped Ratio (%)** | **Unique mapped Reads (%)** | **Multiple mapped Reads (%)** |
| --- | --- | --- | --- | --- |
| Dorsal1 | 36,171,432 | 78.30 | 29,064 (0.06%) | 36,142,368 (78.24%) |
| Dorsal2 | 37,073,292 | 78.90 | 29,780 (0.06%) | 37,043,512 (78.83%) |
| Dorsal3 | 36,261,686 | 77.93 | 33,796 (0.07%) | 36,227,890 (77.86%) |
| Ventral1 | 36,819,864 | 79.05 | 28,670 (0.06%) | 36,791,194 (78.99%) |
| Ventral2 | 35,768,380 | 76.60 | 29,698 (0.06%) | 35,738,682 (76.54%) |
| Ventral3 | 32,429,124 | 79.68 | 27,382 (0.07%) | 32,401,742 (79.61%) |

Multiple mapped reads, the number of reads aligned to multiple locations in the genome;

Unique mapped reads, the number of reads aligned to unique locations in the genome.

**Table S4.** Unigenes annotation.

| **Annotated databases** | **Annotated Number** | **300<=length<1000** | **length>=1000** |
| --- | --- | --- | --- |
| COG | 10,568 | 4,057 | 6,511 |
| GO | 11,685 | 3,687 | 7,998 |
| KEGG | 14,902 | 6,380 | 8,522 |
| KOG | 25,432 | 12,010 | 13,422 |
| Pfam | 24,386 | 9,793 | 14,593 |
| Swiss-Prot | 26,807 | 11,763 | 15,044 |
| eggNOG | 38,156 | 17,755 | 20,401 |
| NR | 40,871 | 19,395 | 21,476 |
| All | 42,866 | 20,792 | 22,074 |

Annotated databases: functional database; Annotated Number: the number of unigenes annotated to the database; 300 < = length < 1000: the number of unigenes annotated to the database with a length greater than 300 bp and less than 1000 bp; Length > = 1000: the number of unigenes with a length greater than 1000 bp annotated to the database.

**Table S6.** Equation and determination coefficient of hormone standard curve.

| **Hormone** | **Equation** | **R-squared** |
| --- | --- | --- |
| IAA | y = 72353.4847x + 2104.9366 | 0.9995 |
| ME-IAA | y = 162944.9838x - 1789.644 | 0.9995 |
| IBA | y = 1814.5872x + 2013.0275 | 0.9989 |
| ICA | y = 141478.4411x + 25456.3847 | 0.9997 |
| IP | y = 470502.2439x - 2817.3904 | 0.9999 |
| tZ | y = 218576.4256x - 542.298 | 0.9999 |
| cZ | y = 275387.9356x - 2031.5008 | 0.9996 |
| DZ | y = 199891.3675x - 633.0979 | 0.9998 |
| MEJA | y = 56501.6584x - 28312.6036 | 0.9985 |
| JA | y = 32783.8583x + 61877.9528 | 0.9966 |
| H2JA | y = 25439.6352x - 3321.2272 | 0.9997 |
| JA-ILE | y = 39995.6311x - 7263.835 | 0.9996 |
| MESA | y = 149.3007x - 1772.9089 | 0.9987 |
| SA | y = 64551.5813x - 16391.1898 | 0.9954 |
| ABA | y = 16001.9955x - 4306.7395 | 0.9967 |
